# uPARAP/Endo180 receptor is a gatekeeper of VEGFR-2/VEGFR-3 heterodimerisation during pathological lymphangiogenesis

**DOI:** 10.1038/s41467-018-07514-1

**Published:** 2018-12-05

**Authors:** Tania Durré, Florent Morfoisse, Charlotte Erpicum, Marie Ebroin, Silvia Blacher, Melissa García-Caballero, Christophe Deroanne, Thomas Louis, Cédric Balsat, Maureen Van de Velde, Seppo Kaijalainen, Frédéric Kridelka, Lars Engelholm, Ingrid Struman, Kari Alitalo, Niels Behrendt, Jenny Paupert, Agnès Noel

**Affiliations:** 1 Laboratory of Tumor and Development Biology, GIGA (GIGA-Cancer), Liege University, B23, Avenue Hippocrate 13, 4000 Liege, Belgium; 2 Laboratory of Connective Tissues Biology, GIGA-Cancer, Liege University, B23, Avenue Hippocrate 13, 4000 Liege, Belgium; 3 Wihuri Research Institute and Translational Cancer Biology Program, Biomedicum Helsinki, University of Helsinki, 00014 Helsinki, Finland; 4 Department of Obstetrics and Gynecology, CHU Liege, 4000 Liege, Belgium; 5 The Finsen Laboratory/BRIC, Rigshospitalet/University of Copenhagen, Jagtvej 124, 2200 Copenhagen, Denmark; 6 Laboratory of Molecular Angiogenesis, GIGA-Cancer, Liege University, B23, Avenue Hippocrate 13, 4000 Liege, Belgium

## Abstract

The development of new lymphatic vessels occurs in many cancerous and inflammatory diseases through the binding of VEGF-C to its receptors, VEGFR-2 and VEGFR-3. The regulation of VEGFR-2/VEGFR-3 heterodimerisation and its downstream signaling in lymphatic endothelial cells (LECs) remain poorly understood. Here, we identify the endocytic receptor, uPARAP, as a partner of VEGFR-2 and VEGFR-3 that regulates their heterodimerisation. Genetic ablation of *uPARAP* leads to hyperbranched lymphatic vasculatures in pathological conditions without affecting concomitant angiogenesis. In vitro, uPARAP controls LEC migration in response to VEGF-C but not VEGF-A or VEGF-CCys156Ser. uPARAP restricts VEGFR-2/VEGFR-3 heterodimerisation and subsequent VEGFR-2-mediated phosphorylation and inactivation of Crk-II adaptor. uPARAP promotes VEGFR-3 signaling through the Crk-II/JNK/paxillin/Rac1 pathway. Pharmacological Rac1 inhibition in *uPARAP* knockout mice restores the wild-type phenotype. In summary, our study identifies a molecular regulator of lymphangiogenesis, and uncovers novel molecular features of VEGFR-2/VEGFR-3 crosstalk and downstream signaling during VEGF-C-driven LEC sprouting in pathological conditions.

## Abstract (web summary)

VEGF-C drives lymphangiogenesis through binding to its receptors VEGFR-2 and VEGFR-3. Here, Durré et al. identify uPARAP/Endo180 as a critical regulator of VEGFR-2/VEGFR-3 heterodimerisation and downstream signaling in response to VEGF-C, and show that uPARAP deletion leads to the formation of hyperbranched vasculatures in pathological conditions.

## Introduction

The process of lymphangiogenesis involves the outgrowth of new lymphatic vessels from pre-existing ones, and it occurs in numerous pathologies including cancer, inflammatory diseases, fibrosis, and graft transplant rejection^1–4^. Despite recent rapid advances in the field of lymphatic vessel biology^5,6^, little is known about the sprouting behavior of lymphatic endothelial cells (LECs). The activation and guidance of specialised LECs at the tip of lymphatic buds are essential to coordinate a proper response to vascular endothelial growth factors (VEGFs) and to form new functional lymphatics^7,8^. VEGFs can be bound by their tyrosine kinase receptors (VEGFR-1 to VEGFR-3), which interact simultaneously with different cell surface molecules that act as co-receptors and auxiliary proteins^9^, including neuropilins (NRP1 or NRP2)^10–12^, integrins^13,14^, ephrin B2^15^ and heparan sulfate proteoglycan^16^. One of the unknown components in VEGFR signaling and biology is how the resulting multiprotein complexes affect the balance between different activated downstream pathways.

Along with a unique role in driving developmental lymphangiogenesis^3,17^, VEGF-C is viewed as the major growth factor that initiates lymphangiogenic sprouting under pathological conditions^6^. In adults, VEGFR-3 is constitutively expressed by LECs and forms homodimers or heterodimers with VEGFR-2 upon VEGF-C stimulation^18,19^. The function of VEGFR-2/VEGFR-3 heterodimers in blood endothelial cells has been extensively studied during angiogenesis. Heterodimers are prominent in tip cells of angiogenic sprouts^20^. The role of VEGFR-2/VEGFR-3 heterodimers is anticipated but poorly documented in lymphangiogenesis. Interestingly, the development of lymphangiectasia in neonates has been shown to require VEGFR-2 and VEGFR-3 as well as to involve heterodimers^21^. In contrast, VEGFR-3 alone drives lymphatic growth in adult mice^21^. These interesting data highlight the complexity of VEGF-C/VEGFR biology with differential effects of VEGF-C on its receptors depending on physio-pathological conditions. These results also suggest that advances in angiogenesis cannot be directly translated to the lymphangiogenic field. It remains unknown how VEGFR-3/VEGFR-2 homodimerisation and heterodimerisation are fine tuned in LECs as well as how they impact signaling events upon VEGF-C stimulation and LEC migration.

The urokinase plasminogen activator receptor-associated protein, uPARAP/Endo180 (*MRC2* gene) (hereafter designated uPARAP), is an endocytic receptor expressed by migrating cells, including cancer cells, macrophages, fibroblasts and endothelial cells^22^. This cell surface molecule has been reported to promote cell invasion through the following mechanisms: (1) matrix remodeling by internalising large fragments of collagen^23^ and routing it to the lysosome for intracellular degradation^23,24^ and (2) cell chemotaxis^25–27^. No uPARAP implication in vascular biology has yet been reported. We hypothesised that uPARAP contributes to LEC migration during lymphangiogenesis by interfering with VEGFR signaling. To address this issue, we investigated the role of uPARAP in LEC migration and sprouting lymphangiogenesis using complementary in vivo and in vitro models.

Here, we show that uPARAP is a negative regulator of VEGFR-2/VEGFR-3 heterodimerisation in LECs. *uPARAP*-deficiency in mice leads to hyper-sprouting lymphangiogenesis in different pathological models. Thus, the present study demonstrates that uPARAP has a gatekeeper function that restricts VEGFR-2/VEGFR-3 heterodimerisation, thereby limiting VEGFR-2-mediated inactivation of Crk-II, an adaptor involved in the JNK/paxillin/Rac1 signaling pathway.

## Results

### *uPARAP* ablation affects pathological lymphangiogenesis

**Fig. 1:**
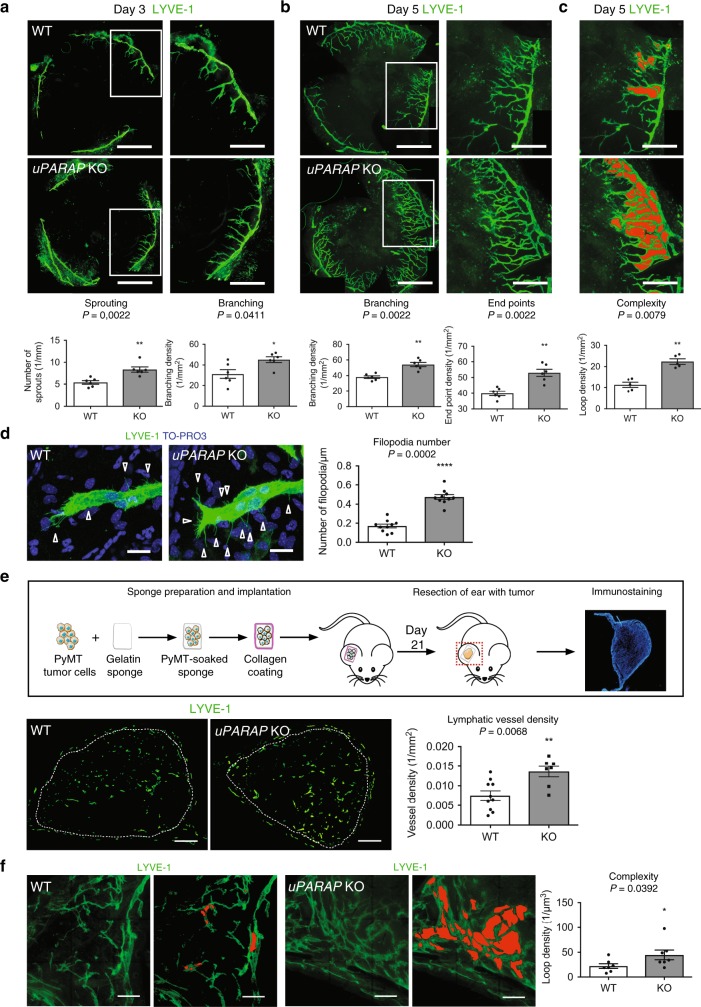
*uPARAP* deficiency induces hyperbranched lymphatic vasculature in cauterised cornea and tumoral lymphangiogenesis. **a**–**c** Whole-mount immunofluorescence of cornea at day 3 (**a**) and day 5 (**b**, **c**) after thermal cauterisation induced in *uPARAP* KO and WT mice (*n* = 6). Bars = 1 mm and 500 µm in the left and right (higher magnification of the insert) images, respectively. Histograms correspond to a computerised quantification of the number of sprouting (**a**, *n* = 6 cornea), branching (**a**, **b** *n* = 6 cornea), end points (**b** *n* **=** 6 cornea) and loop structures (red, **c** *n* = 5 cornea). **d** Filopodial extensions (arrow heads) from lymphatic sprouts. Blue staining corresponds to nuclei detection by TO-PRO3 staining. Bars = 200 µm. Quantification was done on 10 pictures. **e** Schematic of the ear sponge assay using gelatin sponges soaked with PyMT tumor cells. White dots delineate the sponge in the ear. Histogram represents the area density of vessels quantified by a computer-assisted method (*n* = 10 for WT; *n* = 7 for KO). Bars = 500 µm. **f** Vasculature complexity evidenced by loop structures (red) observed on thick sections (150 µm) (*n* = 7). All results are expressed as mean ± SEM, and statistical analyses were performed using a non-parametric Mann–Whitney test. *P < 0.05, **P < 0.01 and ****P < 0.0001

We first assessed lymphangiogenesis in a corneal assay applied to *uPARAP*-deficient (*uPARAP* KO) mice and their wild type (WT) littermates. Three days after cauterisation, a marked increase in the number of vessel sprouting from the limbus was observed in *uPARAP*-deficient mice by LYVE-1 immunostaining (Fig. 1a). Two days later (5 days post-cauterisation), the increased lymphangiogenic response characterised by enhanced vessel branching and end points was maintained in *uPARAP* KO mice, revealing hyperbranched vasculature (Fig. 1b). In WT mice, the lymphatic network harbored mainly a dichotomous branching structure, in which a mother vessel gave rise to two independent daughter branches (Fig. 1c). In sharp contrast, in *uPARAP* KO mice, the lymphatic vasculature displayed an hyperbranched phenotype characterised by a twisted pattern with twice as many loop structures than in WT mice. The computerised quantification performed on whole mounted corneas allowed to discriminate loops from overlapping vessels (Fig. 1c). The number of filopodia at the front of lymphatic sprouts was 2.8-fold more in *uPARAP*-deficient mice than in WT mice (Fig. 1d). Interestingly the overall direction of the tip cell filopodia was impaired in *uPARAP* KO mice. In the absence of uPARAP, tip cell filopodia were not paralleled to the axis of cell migration but rather perpendicular to the cell, suggesting a defect in the ability to sense the pro-lymphangiogenic factor gradient (Fig. 1d). We also utilised the corneal assay to evaluate angiogenesis and lymphangiogenesis. Importantly, the angiogenic response was not affected by *uPARAP*-deficiency, highlighting a specific implication of uPARAP during the pathological lymphangiogenic process (Supplementary Fig. 1a). The recruitment of CD11b-positive cells and F4/80-positive macrophages, which are a major sources of pro-lymphangiogenic factors^4,28^, was not affected by uPARAP status (Supplementary Fig. 1b–e).

To extend our study to tumoral lymphangiogenesis, gelatin sponges populated with murine syngeneic breast tumor cells (FvBn genetic background) were implanted into ears of *uPARAP*-deficient or *uPARAP*-proficient mice (ear sponge assay, Fig. 1e). The density of lymphatic vessel sections was higher in carcinomas developed in *uPARAP* KO mice as compared to their WT counterparts (Fig. 1e). Importantly, the angiogenic response was not impacted by *uPARAP* deletion (Supplementary Fig. 2a). The evaluation of 3D lymphatic architecture was achieved through computer-assisted analysis of 3D image constructions from z-slice images of thick tumor sections. A hyperbranched vasculature with more loop structures was found in *uPARAP* KO mice compared to WT mice (Fig. 1f).

To explore the impact of *uPARAP* ablation on the establishment of normal lymphatic vasculature in mice, we examined the tail dermal lymphatic network that forms during the first postnatal week. Mature superficial dermal lymphatic vessels have a honeycomb pattern, consisting of a hexagonal lattice of capillaries containing a lymphatic ring complex at each junction. No difference in vessel branching density was observed between the two mouse genotypes (Supplementary Fig. 2b). Consistently, in adult ear mice, the lymphatic vessel network was similar in *uPARAP*-deficient and *uPARAP*-proficient mice (Supplementary Fig. 2c). Altogether, these in vivo data highlighted a denser and hyperbranched lymphatic vasculature during the onset of pathological lymphangiogenesis in *uPARAP*-deficient mice, while a normal lymphatic network is formed during postnatal lymphatic development and in adult stages.

### uPARAP regulates VEGF-C-mediated lymphangiogenesis in vivo

**Fig. 2:**
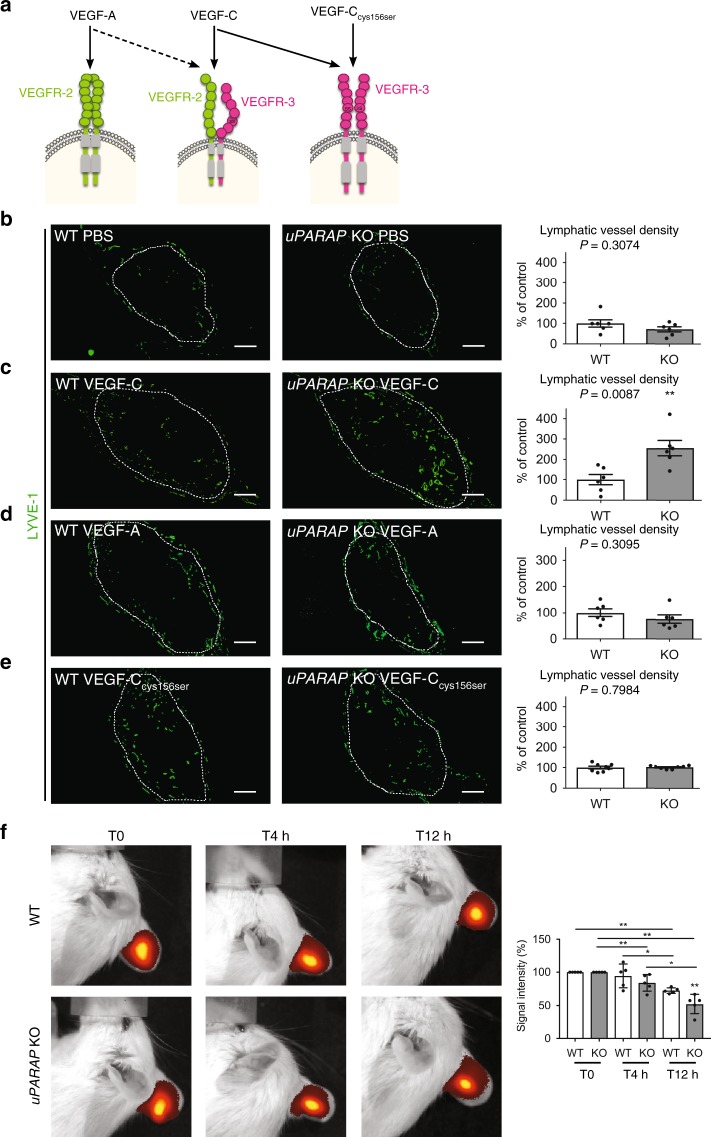
*uPARAP* deficiency stimulates VEGF-C-driven lymphangiogenesis. **a** Schematic of VEGF ligand interaction with VEGFR-2 and VEGFR-3 homodimers or VEGFR-2/VEGFR-3 heterodimers. **b**–**e** Gelatin sponges soaked with PBS (**b**), VEGF-C (**c**), VEGF-A (**d**), or mutated VEGF-C_Cys156Ser_ (**e**) were implanted in mouse ears. White dots delineate the sponge in the ear. Lymphatic vasculature was examined by LYVE-1 (green) immunostaining. Histograms represent the area density of vessels quantified by a computer-assisted method and expressed as percentage of WT control (**b**–**d** *n* = 6; **e** *n* = 8). Bars = 500 µm. **f** Indocyanin Green (ICG) clearance in VEGF-C-soaked sponges in *uPARAP* WT and KO mice using Xenogen IVIS. Histogram represents mean fluorescence signal detected at each time point (*n* = 5). All results are expressed as mean ± SEM, and statistical analyses were performed using a non-parametric Mann–Whitney test. *P < 0.05 and **P < 0.01

To determine the impact of uPARAP on LEC response to specific lymphangiogenic growth factors, we implanted gelatin sponges soaked with the following reagents into mouse ears: PBS, VEGF-C, VEGF-A, or VEGFC_Cys156Ser_ (exclusively binds VEGFR-3 homodimers) (Fig. 2a). After 14 days, the lymphangiogenic response was 2-fold higher in VEGF-C-soaked sponges implanted in *uPARAP* KO mice than in their normal counterpart (Fig. 2c). In sharp contrast, lymphatic vessel density was similar in both genotypes when sponges were soaked with PBS, VEGF-A or VEGF-C_Cys156Ser_ (Fig. 2b, d, e). These data suggested a contribution of VEGFR-2/VEGFR-3 heterodimers in the observed effects resulting from *uPARAP* deficiency. A similar angiogenic response in the different experimental conditions was found (Supplementary Fig. 3), further highlighting a specific contribution of uPARAP in lymphangiogenesis.

We next evaluated lymphatic functionality in the ear sponge assay. Two weeks after the implantation of gelatin sponges soaked with VEGF-C, indocyanine green (ICG) was injected directly into the sponge and its clearance was analysed during 12 h using Xenogen IVIS (Fig. 2f). Importantly, *uPARAP*-deficiency did not result in dysfunctionning lymphatics, but rather to an hyperbranched lymphatic vasculature more efficient in ICG drainage than WT mice. Indeed, 4 h after ICG injection, fluorescent signal was already reduced in *uPARAP* −/− mice, while it remained unchanged in WT mice (Fig. 2f). Later on (after 12 h), ICG signal was 50 and 30% reduced in KO and WT mice, respectively. These data clearly indicate an accelerated clearance of ICG in the absence of uPARAP.

### uPARAP is expressed by human and murine LECs

**Fig. 3:**
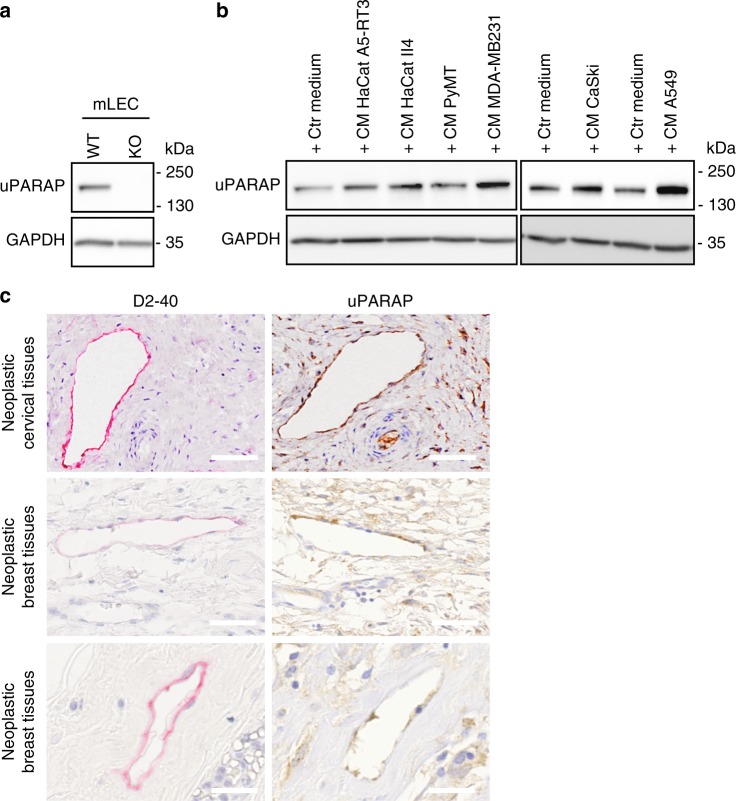
uPARAP is expressed by human and murine LECs. **a** uPARAP expression was assessed by Western Blot on LECs isolated from *uPARAP*-proficient (WT) and deficient (KO) mice. **b** Human LECs were incubated with conditioned media from indicated tumor cell lines and Western Blot of uPARAP was performed. GAPDH was used as a loading control. **c** Immunostainings of lymphatic vessels (pink, D2-40) and uPARAP (brown) on serial sections of human neoplastic cervical and breast tissues. Bars = 50 µm (upper and middle panels) and 25 µm (lower panels)

To demonstrate the expression of uPARAP by murine lymphatic cells, we isolated LECs from WT and *uPARAP* KO mice by flow cytometry (Supplementary Fig. 6a). Western blot analyses confirmed the production of uPARAP by WT LECs, but not by *uPARAP*-deficient LECs (Fig. 3a). In vitro, uPARAP was also expressed by human LECs (HMVEC) under basal conditions and its level of expression was stimulated in the presence of medium conditioned by different tumor cells derived from human skin carcinomas (HaCat A5-RT3, HaCaT II4 cells), murine mammary carcinoma (PyMT cells), human breast carcinoma (MDA-MB231 cells), human cervical carcinoma (CaSki), and human lung carcinoma (A549) (Fig. 3b). The production of uPARAP by LECs in tumors was confirmed by immunohistochemical analyses of human cervical and breast cancer samples (Fig. 3c). The immunostainings using anti-podoplanin (D2/40) and uPARAP antibodies revealed that D2/40 positive lymphatics were stained for uPARAP (Fig. 3c) emphasizing the interest to address the role of uPARAP in LEC functions.

### uPARAP regulates VEGF-C-driven LEC guidance

**Fig. 4:**
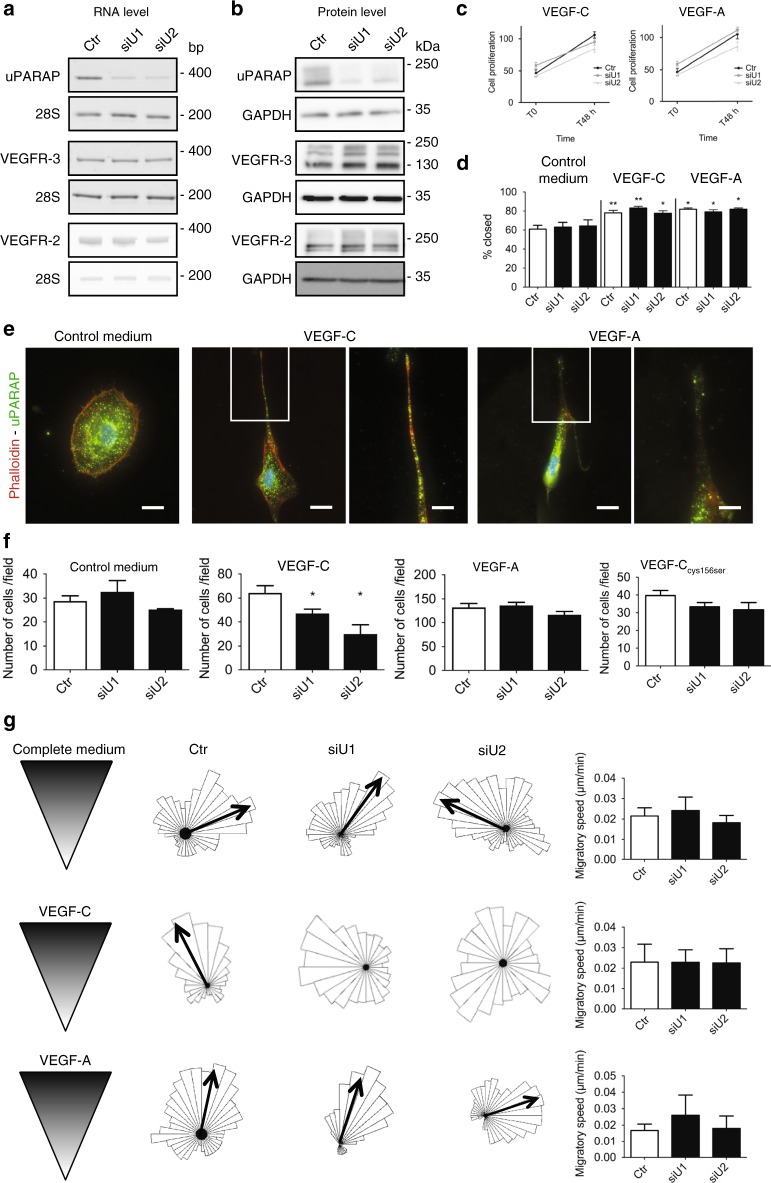
uPARAP downregulation impairs LEC chemotactic migration toward a VEGF-C gradient. LECs were transfected with a siRNA targeting uPARAP (siU1 and siU2) or a control siRNA (Ctr). RT-PCR (**a**) and Western blot (**b**) analyses of uPARAP, VEGFR-3 and VEGFR-2 expression (28S and GAPDH = internal controls). **c** Proliferation upon VEGF-C or VEGF-A stimulation. Data are those of one representative assay out of three (*n* = 12 for T0 and *n* = 8 for T48 h, biological replicates). **d** Scratch assay in the presence of control medium (*n* = 10), VEGF-C (*n* = 10) or VEGF-A (*n* = 3) (biological replicates in all conditions). **e** Phalloidin and uPARAP stainings of LECs migrating toward a VEGF-C or VEGF-A gradient in Ibidi µ-slides. Bars = 25 µm in left images and 50 µm in right images (higher magnification of inserts). **f** Boyden Chamber assay in the presence of control medium, wild type VEGF-C, VEGF-A, or mutated VEGF-C_Cys156Ser_. Histograms correspond to the mean (*n* = 6 for control medium, VEGF-C and VEGF-A, and *n* = 3 for mutated VEGF-C_Cys156Ser_, biological replicates) ± SEM. **g** Chemotactic response to a gradient of complete medium, VEGF-C or VEGF-A. The rose diagram represents the direction of migration of more than 30 cells. Black arrows indicate the mean migration direction. The absence of arrow in VEGF-C diagrams reflects impaired directionality upon uPARAP silencing. Histograms represent the migratory speed (µm/min) where results are expressed as mean (*n* = 6 biological replicates for complete medium and VEGF-C; *n* = 3 biological replicates for VEGF-A) ± SEM. Statistical analyses were performed using a non-parametric Mann–Whitney test. *P < 0.05 and **P < 0.01

We next examined the functional impact of uPARAP silencing on LEC properties in vitro. siRNA-mediated uPARAP downregulation led to at least 80% uPARAP silencing as assessed by RT-PCR and Western blotting (Fig. 4a, b). Such a downregulation of uPARAP did not affect the levels of VEGFR-2 and VEGFR-3 production (Fig. 4a, b). LEC proliferation rate in the presence of VEGF-C or VEGF-A was independent of uPARAP status (Fig. 4c). While uPARAP has been shown to be a regulator of cell migration^25,26^, LEC migration was unchanged upon uPARAP downregulation in scratch assay (Fig. 4d). However, we demonstrated that uPARAP is localised to the leading edge of migrating LECs towards a VEGF-C and VEGF-A gradient (Fig. 4e), nevertheless suggesting a role in cell migration. Indeed, when we analysed the chemotactic properties of LECs, uPARAP silencing drastically impaired the response of LECs to a gradient of VEGF-C, but not VEGF-A or VEGF-C_Cys156Ser_, in a Boyden chamber (Fig. 4f). To further confirm the intrinsic defect in VEGF-C-driven guidance in uPARAP downregulated LECs, we used an additional directional migration assay specifically designed to analyse cell chemotaxis (µ-slide assay) (Fig. 4g). Interestingly, while uPARAP silencing did not influence migration speed (Fig. 4g), the directional migration of LECs was again impaired specifically in a gradient of VEGF-C (Fig. 4g). Taken together, these results demonstrated a specific uPARAP-mediated regulation of VEGF-C-driven cell migration.

### uPARAP restricts VEGFR-2 and VEGFR-3 heterodimerisation

**Fig. 5:**
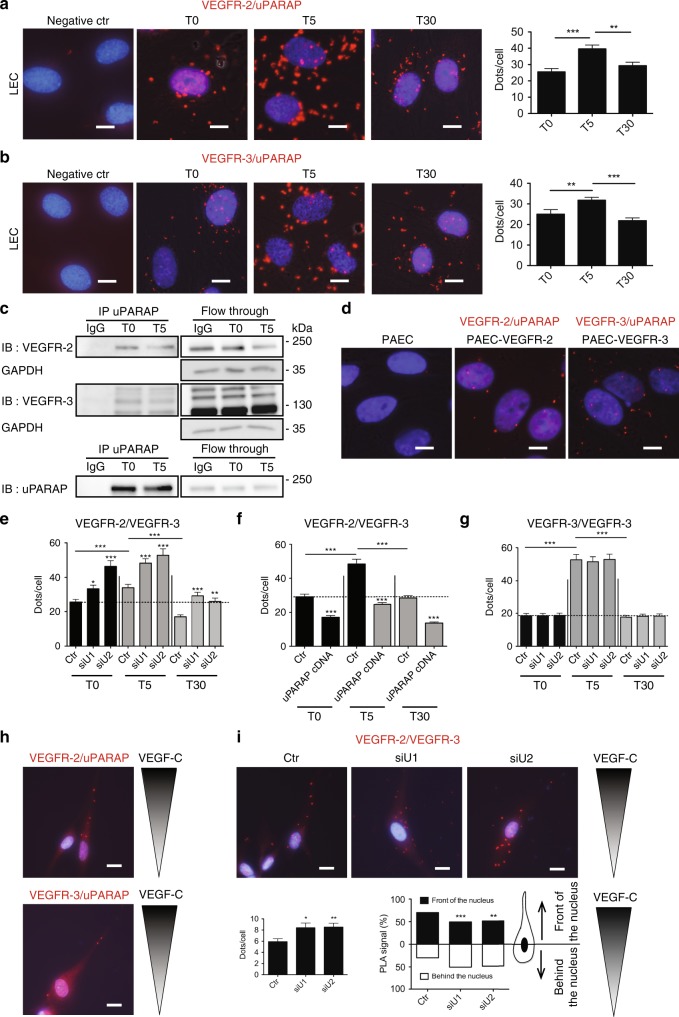
uPARAP forms a complex with VEGFR-2 and VEGFR-3 and restricts VEGFR-2/VEGFR-3 heterodimerisation. In situ PLA in LECs (**a**, **b**, **e**–**i**) or PAECs (**d**) stimulated or not for 5 (T5) and 30 min (T30) with VEGF-C (**a**, **b**, **e**–**g**). **a**, **b** Interaction between uPARAP and VEGFR-2 (**a**) or VEGFR-3 (**b**). Results are those of one representative assay out of 3. Data represent mean (biological duplicates, 20 images analysed for quantification) ± SEM. Bars = 10 µm. **c** Immunoprecipitation (IP) of uPARAP at the indicated time point of VEGF-C stimulation. Western blots were revealed with an antibody (IB) against VEGFR-2, VEGFR-3 (upper panel) or uPARAP (lower panel). GAPDH was used as loading control. **d** In situ PLA detection of uPARAP interaction with VEGFR-2 or VEGFR-3 under basal conditions, in PAECs expressing VEGFR-2 (PAEC-VEGFR-2), VEGFR-3 (PAEC-VEGFR-3) or no VEGFR (PAEC). Bars = 10 µm. **e**, **f** In situ PLA detection of VEGFR-2/VEGFR-3 heterodimerisation in control LECs (Ctr), LECs deficient for uPARAP expression (siU1 and siU2) (**e**), or LECs overexpressing uPARAP (uPARAP cDNA) (**f**). **g** VEGFR-3 homodimerisation in LECs transfected with a VEGFR-3-Flag-tagged cDNA. Data correspond to the mean (biological triplicates, 60 images analysed for quantification, >100 cells analysed per condition) ± SEM (**e**–**g**). **h** In situ PLA detection of uPARAP/VEGFR-2 and uPARAP/VEGFR-3 complexes in LECs migrating in a VEGF-C gradient. Bars = 20 µm. **i** In situ PLA detection of VEGFR-2/VEGFR-3 heterodimers in control LECs (Ctr) and uPARAP silenced LECs (siU) migrating in a VEGF-C gradient. The left histogram corresponds to the mean number of dots per cell. The right histogram represents PLA signal localisation with respect to the nucleus (one representative experiment out of 3 with 25 cells analysed per condition in each experiment). Bars = 20 µm. Statistical analyses were performed using a non-parametric Mann–Whitney test. **i** Chi-square test was used. *P < 0.05, **P < 0.01, and ***P < 0.001

The results observed in the Boyden chamber assay suggest that uPARAP silencing might impact the signaling involving VEGFR-2/VEGFR-3 heterodimer. To determine if uPARAP interacts with one of those receptor, we performed proximity ligation assays (PLA) (Fig. 5a, b). Both types of complexes were observed under basal conditions but were increased after VEGF-C stimulation. The formation of both VEGFR-2/uPARAP and VEGFR-3/uPARAP complexes was increased 5 min after VEGF-C stimulation and reached basal values 30 min later (Fig. 5a, b). Co-immunoprecipitation experiments confirmed the formation of a complex between uPARAP and the two receptors (Fig. 5c). To verify if uPARAP can interact individually with each receptor (VEGFR-2 and VEGFR-3) under basal conditions, we used porcine aortic endothelial cells (PAECs) that do not express these receptors. After cell transfection with cDNA, either VEGFR-2 (PAEC-VEGFR-2) or VEGFR-3 (PAEC-VEGFR-3), PLA confirmed the formation of VEGFR-2/uPARAP and VEGFR-3/uPARAP complexes in cells expressing only one of the two receptors (Fig. 5d). Importantly, siRNA-mediated uPARAP silencing in LECs led to increased VEGFR-2/VEGFR-3 heterodimerisation in basal conditions and after VEGF-C stimulation (Fig. 5e, Supplementary Fig. 4a). Strikingly, increased PLA signals were still detected upon uPARAP silencing 30 min after VEGF-C stimulation, suggesting sustained receptor heterodimerisation. In contrast, overexpression of uPARAP in LECs reduced VEGFR-2/VEGFR-3 heterodimerisation upon VEGF-C stimulation (Fig. 5f, Supplementary Fig. 4b). These data indicated that uPARAP interacts with each VEGF receptor and restricts VEGFR-2/VEGFR-3 heterodimer formation. In sharp contrast, uPARAP silencing did not affect VEGFR-3 homodimerisation as assessed in LECs transfected with VEGFR-3 Flag-Tagged cDNA (Fig. 5g, Supplementary Fig. 4c).

We next analysed the subcellular localisation of uPARAP/VEGFR-2, uPARAP/VEGFR-3, and VEGFR-2/VEGFR-3 complexes in cells migrating in a gradient of VEGF-C. In those conditions, uPARAP/VEGFR-2 and uPARAP/VEGFR-3 complexes were detected in the leading edge of migrating cells (Fig. 5h). Interestingly, in the presence of uPARAP, VEGFR-2/VEGFR-3 heterodimers were also mainly detected in the leading edge of migrating cells. In sharp contrast, upon uPARAP silencing, heterodimers were spatially redistributed as determined by a computerised method (Fig. 5i). Indeed, heterodimers were mainly localised in front of the nucleus in cells expressing uPARAP and migrating in a gradient of VEGF-C (70% of heterodimers localised in front of the nucleus). Upon uPARAP silencing, heterodimers were randomly positioned with respect to the nucleus. Altogether, we provide evidence that uPARAP controls the formation and spatial distribution of VEGFR-2/VEGFR-3 heterodimers in migrating cells.

Given the known endocytic function of uPARAP, we verified whether uPARAP silencing affects or not the availability at the cell surface and the internalisation of both receptors. LECs silenced or not for uPARAP were subjected to flow cytometry (FACS) analyses (for VEGFR-2 and VEGFR-3) performed under non-permeabilised conditions, at different times following VEGF-C stimulation. VEGFR-2 and VEGFR-3 amounts initially detected at the cell surface (at T0) were similar in LECs silenced or not for uPARAP (Supplementary Fig. 5). Therefore, the enhanced heterodimerisation does not rely on increased availability of both receptors at the cell surface. Upon VEGF-C stimulation, both receptor amounts started to decrease after 5 min, reaching very low levels after 30 min of stimulation. The internalisation curve of each receptor was similar and independent of uPARAP status (Supplementary Fig. 5).

### uPARAP controls the Crk-JNK-paxillin-Rac1 pathway

**Fig. 6:**
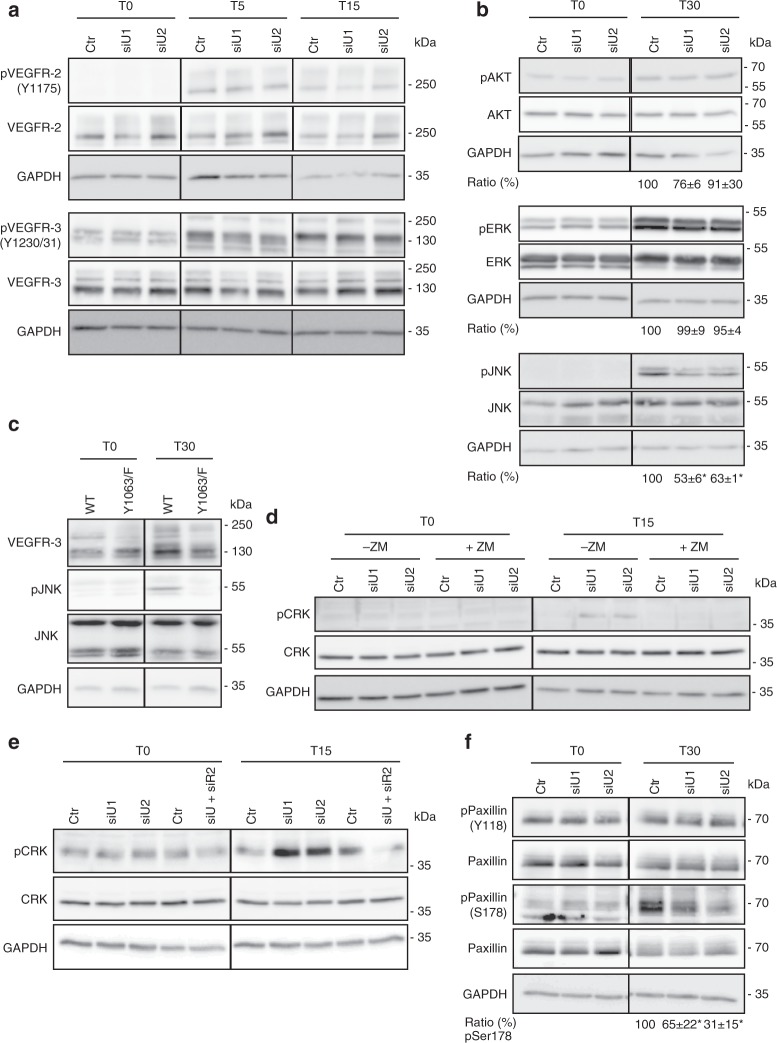
uPARAP downregulation impairs the Crk/JNK/paxillin pathway. **a**, **b**, **d**–**f** LECs were treated with siRNAs targeting uPARAP (siU1 and siU2) or a control siRNA (Ctr) and stimulated for the indicated time (minutes) with VEGF-C. **a**–**f** Western blot of total and phosphorylated (p) VEGFR-2 (Y1175), VEGFR-3 (Y1230/31) (**a**), ERK, AKT, JNK (**b**, **c**), Crk (**d, e**) and Paxillin (Y118, S178) (**f**). **c** PAECs expressing uPARAP were transfected with a plasmid carrying an intact (WT) or mutated (VEGFR-3_Y1063/F_) VEGFR-3 cDNA. JNK phosphorylation was evaluated by Western blot after VEGF-C stimulation. **d**, **e** LECs were treated (+ZM) or not (-ZM) with VEGFR-2 inhibitor (ZM323881), or siRNA against VEGFR-2 (siR2). For quantification of Western blot band densities, values were calculated as the ratio of phosphorylated to total form and normalised to the values obtained with the control (Ctr) (*n* = 3, biological replicates). Statistical analyses were performed using a non-parametric Mann–Whitney test. *P < 0.05

**Fig. 7:**
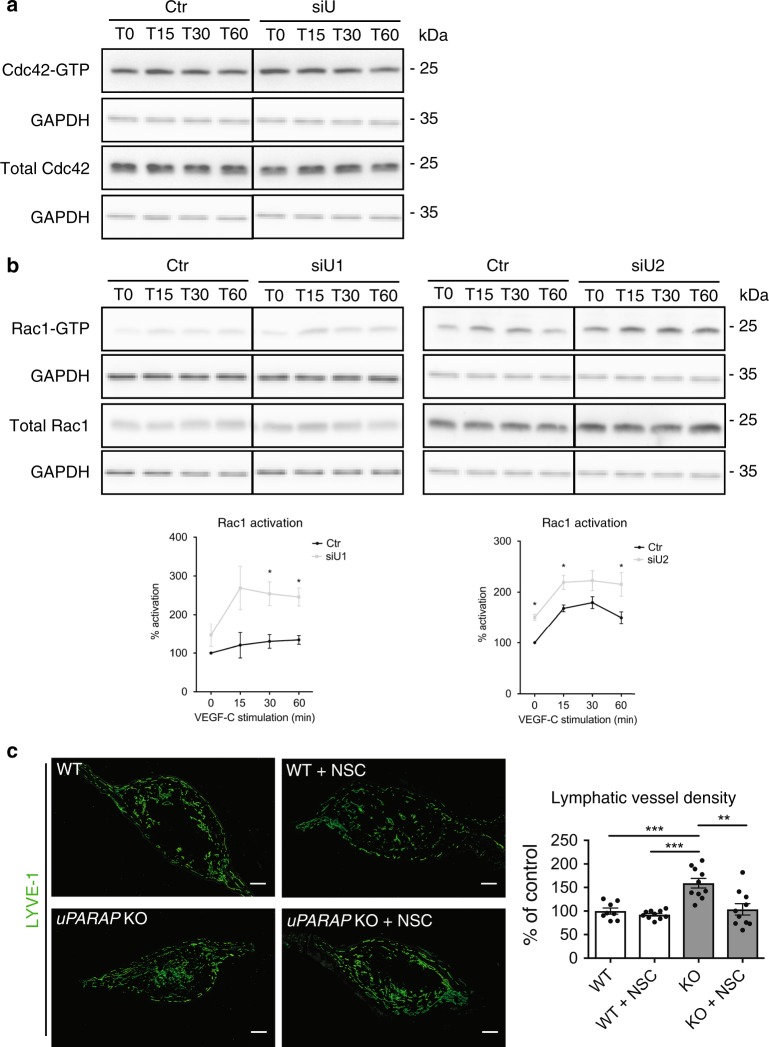
uPARAP downregulation induces Rac1 over-activation upon VEGF-C stimulation. **a**, **b** Cdc42 (**a**) and Rac1 (**b**) time course activation in uPARAP-silenced (siU) or control (Ctr) LECs. For pull-down quantification, values were calculated as the ratio of Rac1-GTP to total Rac1 form and normalised to the values obtained with the control (Ctr) (*n* = 4, biological replicates). **c** Gelatin sponges soaked with VEGF-C were implanted in mouse ears. Sponges were injected or not with Rac1 inhibitor, NSC 23766 (NSC). Lymphatic vasculature was examined by LYVE-1 (green) immunostaining. Bars = 500 µm. Histogram represents the area density of vessels quantified by a computer-assisted method and expressed as percentage of WT control. Values correspond to the mean (*n* = 10) ± SEM. Statistical analyses were performed using a non-parametric Mann–Whitney test. *P < 0.05, **P < 0.01, and ***P < 0.001

Importantly, uPARAP downregulation did not affect the phosphorylation of VEGFR-2 (Y1175), VEGFR-3 (Y1230/31), ERK or AKT upon VEGF-C stimulation (Fig. 6a, b). In sharp contrast, this silencing decreased JNK phosphorylation 30 min after VEGF-C treatment (Fig. 6b). Similarly, lower JNK phosphorylation levels were found in VEGF-C-stimulated LECs issued from *uPARAP* KO mice (Supplementary Fig. 6b). Given the reported implication of Y1063 of VEGFR-3 in JNK phosphorylation^29,30^, we generated a mutated form of VEGFR-3 with a substitution of Y1063 with Phe (VEGFR-3_Y1063/F_) by site-directed mutagenesis. PAECs were transfected with WT or VEGFR-3_Y1063/F_ cDNA. Interestingly, this single Y1063→F substitution was sufficient to abrogate VEGF-C-induced JNK phosphorylation, thereby underlying the importance of this tyrosine residue (Fig. 6c). Tyr1063 can serve as a docking site for the C10 regulator of kinase-II (Crk-II, herein referred as Crk) adaptor involved in JNK activation^29^. uPARAP silencing increased Crk phosphorylation, which involved VEGFR-2 as assessed by the use of a pharmacological inhibitor (ZM323881) and siRNA (Fig. 6d, e). Crk phosphorylation is known to induce intramolecular binding with its SH2 domain, thereby lowering its interaction with its partners. The downregulation of uPARAP also led to reduced phosphorylation of paxillin, another known Crk partner and downstream target of JNK pathway (Fig. 6f). Of note, only phosphorylation at Ser178, a site targeted by JNK^31^, was modulated by uPARAP silencing, while JNK-independent Tyr phosphorylation (Y118) was unchanged (Fig. 6f). Having established a link among JNK, paxillin and Crk in LECs (Fig. 6b–f), we next evaluated downstream effectors involved in cell migration. Pull-down assays were performed to analyse the temporal activation of small Rho GTPases (Rac1 and Cdc42), known regulators of cell migration whose activity can be indirectly controlled by paxillin^32^. Upon uPARAP silencing, higher amounts of GTP-bound Rac1 were detected after VEGF-C stimulation, revealing an overactivation of Rac1 (Fig. 7b). No difference in Cdc42 activation was observed (Fig. 7a). For in vivo validation of Rac1 contribution, a pharmacological Rac1 inhibitor (NSC 23766) was locally applied to ear sponges soaked with VEGF-C and implanted in *uPARAP*-proficient and *uPARAP*-deficient mice (Fig. 7c). NSC 23766 had no effect on lymphangiogenesis in WT mice. Notably, in *uPARAP*−/− mice, Rac1 inhibition restored the lymphangiogenic response to a level similar to that observed in WT mice, demonstrating the contribution of Rac1 in the exacerbated lymphangiogenic response. Altogether, our data indicated that uPARAP silencing attenuates JNK activation and modulates the functions of a scaffold (paxillin) molecule leading to the over-activation of Rac1 that can contribute to the hyperbranching phenotype observed in *uPARAP*-deficient mice.

## Discussion

**Fig. 8:**
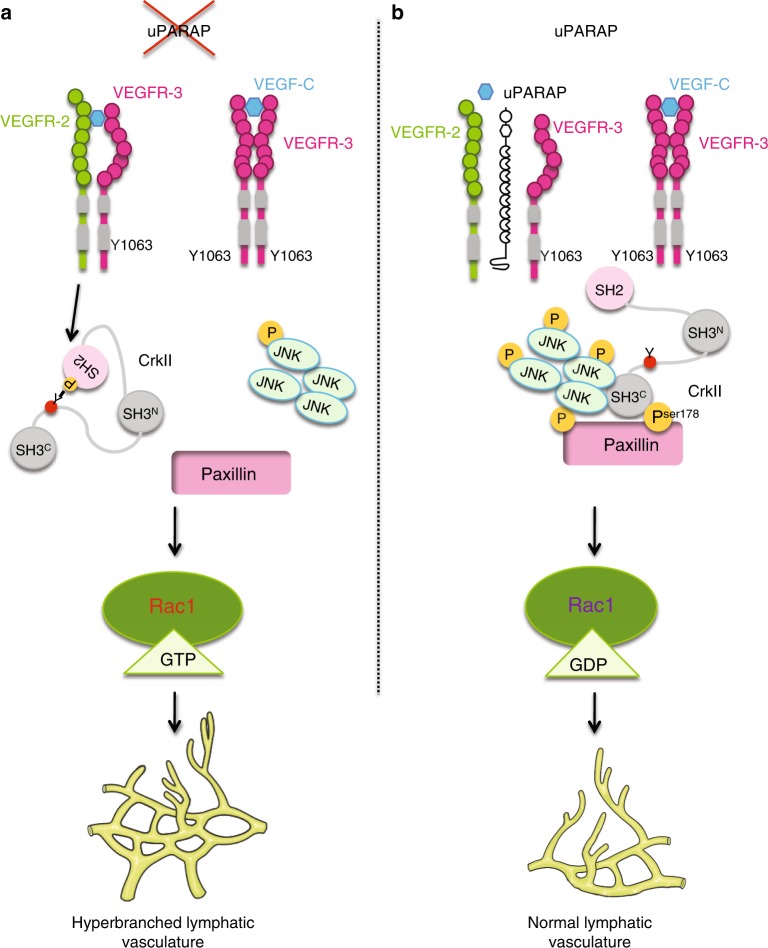
Schematic representation of the proposed model. **a** In the absence of uPARAP, VEGFR-2/VEGFR-3 heterodimerisation is promoted leading to Crk phosphorylation by VEGFR-2, which impairs its interaction with its partners (JNK and paxillin). Hyperbranched lymphatic vasculature is observed in the absence of uPARAP in vivo. **b** uPARAP acts as a gatekeeper, restricting VEGFR-2/VEGFR-3 heterodimerisation and the subsequent phosphorylation of Crk, which acts as a bridge between partners enhancing JNK signaling

The present study addressed novel features of VEGF-C signaling in LECs. This study identified a specific role for uPARAP during lymphangiogenesis, which was supported by in vivo genetic ablation in mice and in vitro silencing approaches. Interfering with uPARAP expression resulted in functional hyperbranched lymphatic vasculature in pathological conditions in vivo and impaired directional migration of LECs in vitro under VEGF-C stimulation. Mechanistic investigations provided the following conclusions: (i) uPARAP is found in molecular complexes with VEGFR-2 and VEGFR-3, (ii) uPARAP restricts VEGFR-2/VEGFR-3 heterodimerisation and controls the spatial distribution of heterodimers, (iii) uPARAP promotes the inactivation of Crk by VEGFR-2-mediated phosphorylation, and (iv) uPARAP tightly regulates Rac-1 activation via the VEGFR-3_Y1063_/JNK/paxillin pathway (Fig. 8).

In the present study, we made four important discoveries that give mechanistic insights into sprouting lymphangiogenesis under pathological conditions. The most prominent finding in this report was the discovery of uPARAP function in lymphatic sprouting. In mice, under challenging conditions, *uPARAP* gene ablation resulted in a denser and hyperbranched lymphatic vasculature displaying more vascular loops. These observations were consistent in tumor xenografts and corneal lymphangiogenesis induced by thermal cauterisation. We showed that uPARAP is essential for an adequate response of LECs to VEGF-C stimulation both in vivo (in the sponge assay) and in vitro (in two models of directional migration). The implication of uPARAP in lymphangiogenesis has not been previously reported and was not anticipated. The relevance of our finding is supported by the detection of uPARAP in lymphatic vessels of human cancers. Interestingly, the effect of uPARAP is specific to lymphangiogenesis because no modulation of the concomitant angiogenic response was noted in the corneal and ear sponge assays. This was an intriguing finding because uPARAP is expressed both by LECs and blood endothelial cells (BECs), such as HUVECs^33^. Although pathway-related molecules of the VEGF-C/VEGFR-3 axis are not restricted to LECs, VEGFR-3 is highly expressed by LECs. Therefore, the implication of uPARAP in LEC properties is likely related to the availability of VEGFR-2/VEGFR-3, of which expression levels differ in LECs and BECs and may vary in physiological and pathological conditions. Another interesting finding is that *uPARAP* deficiency did not affect tail and ear lymphatic networks in homeostatic conditions. These findings suggested a specific effect of uPARAP on the directional migration of LECs during pathological lymphangiogenesis. Nevertheless, we cannot exclude the possibility that uPARAP affects specific steps of lymphatic development such as for instance, the budding of Prox-1 positive endothelial cells from cardinal vein, the organisation of primitive sacs, veinus endothelial cells differentiation into LECs, recruitment of progenitor LECs and/or vessel organisation at latter stages. The clear contribution of uPARAP in pathological but not in developmental lymphangiogenesis could be related to inflammation associated to post-developmental lymphangiogenesis that results in different levels of various growth factors and cytokines/chemokines. It may also depend on the contextual balance between VEGFR-2, VEGFR-3 and their heterodimer. Notably, different levels of VEGFR-2/VEGFR-3 heterodimers were found in adult and neonates resulting in different sensitivity to VEGF-C induced pulmonary lymphangiectasia^21^.

The second important discovery was that uPARAP controls VEGFR-2/VEGFR-3 heterodimerisation without affecting the internalisation of those receptors. This conclusion was first deduced from the use of the VEGF-C_Cys156Ser_ mutant, a specific VEGFR-3 ligand. Through PLA, we provided in situ evidence that uPARAP prevents heterodimer formation and regulates their spatial distribution in LECs migrating in a VEGF-C gradient. Surprisingly, receptor heterodimerisation observed in absence of uPARAP was not strictly ligand dependent. Indeed, receptor heterodimerisation was already detected under basal conditions and further increased upon VEGF-C stimulation. This finding indicated that receptor heterodimerisation is not only guided by the presence of growth factors but also by the availability of an auxiliary protein. Interestingly, uPARAP silencing did not affect VEGFR-3 homodimerisation. It is worth noting that VEGFR-2 activation by VEGF-C has been reported in vitro, in PAE cells expressing only VEGFR-2^34^. However, when both receptors are endogenously expressed in blood endothelial cells, VEGF-C stimulation only induced the formation of VEGFR-2/R-3 heterodimers and VEGFR-3 homodimers whereas VEGFR-2 homodimers were not detected in vitro^20^. An additive effect of uPARAP on VEGFR-2 activation in homodimers is thus unlikely.

The underlying mechanisms by which uPARAP controls the extent of VEGFR-2/VEGFR-3 complex formation may involve blockade by steric hindrance. Although crystal structure analyses have underlined the implication of membrane proximal Ig homology domains of VEGFR-2^35^ and VEGFR-3^36^ for their respective dimerisation, no data are available regarding the VEGFR-2/VEGFR-3 complex to the best of our knowledge. Each VEGFR may undergo conformational changes when engaged in a complex with uPARAP, and certain sites may be hidden or inaccessible for the other molecule. Alternatively, uPARAP may recruit or exclude additional molecular mediators that interfere with VEGFR-2/VEGFR-3 complex formation. VEGF receptors are known to interact with different cell surface molecules, including integrins^13,14^, proteoglycans^16^ and co-receptors, such as semaphorins, ephrins and neuropilins^10–12,15,37^. Altogether, uPARAP interferes with the interaction between VEGFR-2 and VEGFR-3, and it may offer a way for LECs to fine-tune their response to different growth factors.

The third key finding relied on the identification of an unusual downstream signaling pathway modulated by uPARAP silencing (Fig. 6). Deng et al.^5^ previously reported that VEGFR-2/VEGFR-3 complex formation is crucial to VEGF-C-induced AKT activation, whereas ERK activation is mainly driven by the VEGFR-3 homodimer. In the present study, uPARAP down-regulation did not affect ERK or AKT phosphorylation. Unexpectedly, JNK phosphorylation and paxillin phosphorylation at Ser178, rather than Tyr118, was reduced upon uPARAP silencing. Through site-directed mutagenesis of VEGFR-3, we confirmed the role of tyrosine residue 1063 of VEGFR-3 in JNK phosphorylation^29^. Furthermore, the observed modulation of paxillin phosphorylation at Ser178 residue was also in line with JNK contribution^31^. Paxillin is a scaffold protein that can exert different effects, including the recruitment of regulators (activator and/or inhibitors) of small RhoGTPase activity^32^. Interestingly, Rac1 overactivation was detected in uPARAP-silenced LECs after VEGF-C stimulation. Notably, the pharmacological inhibition of Rac1 in *uPARAP*-deficient mice restored a lymphangiogenic response to a similar level to that in WT mice. Such a regulatory role of uPARAP in small RhoGTPase activation was in agreement with previous studies, which reported that uPARAP regulates tumor cell mobility (MCF7 and MDA-MB231) induced by urokinase (uPA) and growth factors via the regulation of Cdc42 and Rac1 activation^25,26^. In our study, however, only Rac1 activation was affected by altered uPARAP expression. Another difference with this previous work was that uPARAP downregulation did not affect the speed of LEC migration in response to VEGF-C, while a drastic reduction of tumor cell velocity has been reported upon uPA or growth factor stimulation^25^. Altogether, these data suggested cell-specific regulation by uPARAP.

The fourth discovery was that the VEGFR-2/VEGFR-3 heterodimer negatively controls the VEGF-C/VEGFR-3/JNK pathway. Using a pharmacological and siRNA approaches, we provided evidence that VEGFR-2 contributes to Crk inhibition by phosphorylation, which agreed with previous data^38^. Crk is an adaptor, which interacts with phosphorylated tyrosine residues of VEGFR by its SH2 domain and with downstream partners, including JNK and paxillin, through its SH3 domains^29^. Our data supported the concept that VEGFR-2 activated by VEGF-C can exert a negative regulation on VEGF-C/VEGFR-3 signals (Fig. 6). It is likely that this negative regulation of the VEGFR-3 pathway by VEGFR-2 is rather weak due to the restriction in VEGF-C-induced receptor heterodimerisation imposed by uPARAP.

Altogether, the present study shed light on VEGF/VEGFR biology and assigned an unexpected function to uPARAP during pathological VEGF-C-induced lymphangiogenesis. In addition to identifying a molecular regulator of lymphangiogenesis, our study uncovered molecular features of VEGFR-2/VEGFR-3 crosstalk and downstream signaling during VEGF-C-driven LEC sprouting in pathological conditions. Importantly, we provide the first evidence that a finely balanced heterodimer/homodimer ratio regulates lymphatic vessel branching and functions. Given the importance of lymphatic vessels in fluid drainage as well as in antigen and cell transport from the interstitial space to lymph nodes, our findings have important implications in terms of lymphatic functionality. Our data suggest that developing strategies interfering with VEGFR-2/VEGFR-3 heterodimerisation may be therapeutically useful to overcome lymphatic vasculature dysfunctionality or improve lymphatic vessel function, such as for instance in the case of lymphedema. Experiments addressing how uPARAP and VEGFR-2/VEGFR-3 signaling influence vessel functionality and cell transport will undoubtedly enhance our understanding of lymphatic functional regulation under physiological and pathological conditions.

## Methods

### Cells and reagents

Primary human LECs (HMVEC-dLy from Lonza (CC-2810) or HDLEC adult from PromoCell (C-12217)) were cultured in EGM2-MV medium (herein referred as complete medium) (CC-3202, Lonza) until confluence. LEC transfections with siRNAs targeting uPARAP (siU1, 5′ CCCAACGUCUUCCUCAUCU 3′; and siU2, 5′ GGGCUGUACCUACGUAGAU 3′, Eurogentec), and VEGFR-2 (5′ ACAAUGACUAUAAGACAUGCUAUGG 3′, IDT-DNA) or with control siRNAs (Ctr, 5′ UGACUGAGUGCGAUCAUGA 3′; Eurogentec, and 5′ CUUCCUCUCUUUCUCUCCCUUGUGA 3′, IDT-DNA) were performed using Interferin according to manufacturer’s recommendations (409-50, Polyplus). LECs were stimulated with recombinant human VEGF-A (10 ng/ml, 293-VE), human VEGF-C (400 ng/ml, 2179-VC), or human VEGF-C_Cys156Ser_ (500 ng/ml, 752-VC), which binds exclusively to VEGFR-3 (all from R&D Systems), in serum-free EBM-2 medium (CC-3156, Lonza). In Fig. 6, serum-starved LECs were treated with (+ZM) or without (−ZM) 10 µM ZM323881 (2475, Tocris). Tumor PyMT cells were derived from Polyoma Middle T (PyMT) transgenic mice^39^. In some assays, porcine aortic endothelial cells (herein referred as PAECs)^40^ were transfected with VEGFR-3 WT or mutant VEGFR-3 cDNA with a Tyr 1063 Phe substitution (VEGFR-3_Y1063F_). PAEC transfections with VEGFR3-WT or VEGFR-3_Y1063F_ cDNA were performed using XtremGene9 according to manufacturer’s instructions (6365779001, Sigma-Aldrich). LECs were transfected with uPARAP cDNA^33^ for uPARAP overexpression assays and with VEGFR-3 Flag-tagged cDNA (HG10806-NF, Sino Biological) for homodimers PLA assays using Viromer Yellow (VY-01LB, Lipocalyx) as transfection reagent.

### Conditioned medium of tumor cells

The different tumor cells used were derived from human skin carcinomas (HaCat A5-RT3, HaCaT II4 cells)^41^, murine mammary carcinoma (PyMT cells)^39^, human breast carcinoma (MDA-MB231 cells)^42^, human cervical carcinoma (CaSki)^43^ and human lung carcinoma (A549)^42^. All these cell lines were negative for mycoplasma contamination. For the preparation of medium conditioned (CM) by tumor cells, subconfluent cells were incubated in serum-free DMEM medium (10938-025, ThermoFisher). After 48 h, CM was collected, centrifuged at 1000×*g* for 10 min, and concentrated 10× with Amicon Ultra Centrifugal Filters 10 K (UFC900324, Millipore). CM aliquots were stored at − 20 °C until use. LECs were stimulated with CM diluted 10× in fresh EBM-2 for 24 h.

### Mice

*uPARAP* −/− or + / + FVB/N mice^44^ (8 to 10-week-old) were used throughout this study, except for tail preparation conducted during the first post-natal week (at P6). The animals were maintained under a 12-h light-dark cycle with free access to food and water. Animal experiments were performed in compliance with the Animal Ethical rules of the University of Liège (Liège, Belgium) after approval of the local Animal Ethical Committee.

### In vivo corneal assay

Corneal lymphangiogenesis was induced by thermal cauterisation, and corneas were whole mounted for immunostainings as previously described^45–47^. To visualise lymphatic vessels, corneas were fixed in 70% ethanol at room temperature for 1 h and then blocked with 3% milk, 3% BSA for 1 h at room temperature. Corneas were then incubated with a polyclonal goat anti-mouse lymphatic vessel endothelial receptor-1 (1/200; AF2125, R&D Systems) antibody overnight at 4 °C. After washing in PBS, an alexa Fluor 488-coupled rabbit anti-goat antibody (1/200; A21222, Invitrogen)^47^ was added for 2 h at room temperature. Double immunostainings with LYVE-1 (1/200; R&D Systems) and CD11b (1/250; 557395, BD Biosciences), F4/80 (1/100; Ab 16911, Abcam) or CD31 (1/200; 553370, BD Biosciences) were performed as previously described by Detry et al.^28^ The lymphangiogenic responses were analysed using a described computerised method^28,45^. All results were normalised to the total cornea area and are expressed as densities. Neovessels were isolated from cornea images, and vascular loops were identified by hole detection algorithms. Loop density was expressed as a number of loops by unit area of the total cornea area. For filopodia observation, images were obtained on a confocal LEICA SP2 at 63× magnification (zoom X2). Nuclei are indicated by blue staining with TO-PRO-3 iodide (1/250; T3605, Invitrogen). The number of filopodia was determined over 75 µm from the tip of lymphatic vessels.

### Tail and ear whole mount preparations

Tail and ear dermal sheets were prepared as previously described^47^. In brief, ears were cut, dorsal halves were separated from the cartilage-containing ventral ear halves and floated epidermal side up on 0.5% ammoniumthiocyanate (A7149, Sigma-Aldrich) solution for 20 min at 37 °C. Epidermis and dermis were separated and fixed in 70% ethanol. The same procedure was applied to collect the dermis of the tail. Lyve-1 staining was performed as described for cornea. Vessel and branching densities were determined with a computerised method (at least 5 images per condition; *n* ≥ 3)^47^.

### Sponge assay with growth factors or tumor cells

Gelatin sponges (Gelfoam, Pfizer) were cut into small pieces (3 mm^3^) and incubated for 30 min in PBS supplemented with recombinant human VEGF-C (1 µg/sponge), human VEGF-A (25 ng/sponge) or human VEGF-C_Cys156Ser_ (1 µg/sponge) before embedding in interstitial type I collagen gel (1.5 mg/ml; 47,254.02, Serva). In some assays, PyMT tumor cells were incubated with sponges (500,000 cells/sponge) for 30 min in EBM-2 serum-free medium. After 14 or 21 days of transplantation into mouse ears, sponges were removed and embedded in tissue OCT (00411243, VWR). For lymphatic and blood vasculature immunostaining, sections of 10 µm were fixed in 4% formol (11699408, VWR) for 10 min and permeabilised for 5 min with 1% Triton X-100 (108603, Millipore) at room temperature. Sections were blocked for 20 min in Animal-Free blocking solution (15019 L, Cell signaling) and incubated for 2 h at room temperature with CD31 (1/200; ab.28364, Abcam) and LYVE-1 (1/200; AF2125, R&D Systems) antibodies. After washing in PBS, Donkey anti-rabbit Alexa Fluor 555 (1/200; A31572, Invitrogen) and Donkey anti-goat Alexa Fluor 488 (1/200; A11055, Invitrogen) were added for 30 min at room temperature. At least 30 images per experimental condition were used for computerised quantification^48^. For 3D analysis, 150-µm-thick sponge slices were cut, immunostained and imaged with a Zeiss confocal microscope LSM 880 with Airyscan at 10× magnification. Images of several millimeters wide were obtained by image stitching. They were analysed by automatic computerised methods adapted from 2D corneal assays to 3D images. Loop density is expressed as a number of loops by unit volume of the total vessels contained in the sponge. For Rac1 inhibition experiment, Rac1 inhibitor NSC 23766 (66 µg per sponge; 553502, Millipore) was injected directly in sponges twice a week with an Hamilton syringue. After 14 days of treatment, sponges were harvested and analysed as described above^49^.

### Indocyanine green clearance

Mice were anaesthetised with 2% isoflurane (B506, Zoetis). The mice were then positioned inside an IVIS 200 Vivo Vision (Xenogen, Caliper Life Sciences) on their ventral side, and precontrast injection images were taken to establish background signal intensities at tissues of interest. The imaging parameters were as follows: λex = 745 nm, λem = 840 nm, exposure time = 1 s, f/stop = 2, small binning, field of view = 6.6 × 6.6 cm^2^. Four microliters of indocyanin green dye (Verdye 0.1 mg/ml) were injected at the top of the ear sponge. Images were acquired immediately after injection and every 60 min thereafter. For image analysis, Living Image software (Caliper Life Sciences) was used. Regions of interest (ROI) were placed over the ear. Average signal intensity values were recorded for each ROI and plotted versus time in GraphPad Prism.

### Fluorescence-activated cell sorting (FACS)

LECs were isolated from adult murine lungs using cell sorting. Briefly, mice were sacrificed, lungs were dissected and flushed with ice-cold HBSS (14185-045, Gibco) and cut into 1 cm pieces. The tissue was then digested with a solution containing 0.04% Dispase II (25766700, Sigma-Aldrich), 0.25% Collagenase II (LS004176, Worthington Lab) and 0.01% DNase I (11284932001, Sigma-Aldrich) in sterile PBS for 30 min at 37 °C and washed with complete medium. Cell suspension was centrifuged, washed with PBS, blocked with PBS 5% FBS and then incubated with labeled antibodies (each incubation for 20 min on ice): Podoplanin-AlexaFluor 488 (1/200; 127406), CD31-APC (1/80; 102510), CD45-Brilliant Violet 421 (1/40; 103134, all from Biolegend). FACS sorting was performed on a BD FACSAria III (BD Biosciences).

To analyse cell surface receptors, FACS experiments were performed on LECs detached with Accutase (SCR005, Millipore) as previously described^50^. For cell surface staining, non-permeabilised conditions were used. After blocking with 10% normal goat serum (NGS) and 2% bovine serum albumin (BSA) in PBS for 30 min, cells were incubated for 1 h at 4 °C with a mouse anti-VEGFR-3 (1/100; MAB 3757, Millipore) or a mouse anti-VEGFR-2 (1/100; MAB 3571, R&D Systems) antibodies in PBS containing 0.5% BSA and 2% NGS. After washing with PBS containing 0.5% BSA and 2% NGS, cells were incubated for 30 min at 4 °C with Alexa Fluor 488-coupled goat anti-mouse IgG (1/200; A 11029, Life Technologies) for VEGFR-3, or with Polyclonal goat anti-mouse Immunoglobulins/Biotinylated (1/100; E 0433, Dako) and APC Streptavidin (1/100; 554067, BD Biosciences) for VEGFR-2. Cells were analysed in a FACSCanto II flow cytometer (Becton Dickinson) and graphs were performed using FlowJo Software.

### RT-PCR

Total LEC RNA was isolated using the High Pure RNA isolation Kit (11828665001, Roche). RT-PCR was performed with 10 ng of total RNA with the GeneAmp® Thermostable rTth Reverse Transcriptase RNA PCR Kit (Applied Biosystems). RT-PCR products were observed after electrophoresis in 10% acrylamide gels and staining with Gel Star (50535, Lonza). The following primers were used: 5′-TGTCAGTCAGCTGGTCCTTG-3′ (reverse primer) and 5′-CCTGGGCATGTATGAGTGTG-3′ (forward primer) for uPARAP; 5′-TGCAGAACTCCACGATCACC-3′ (reverse primer) and 5′-CCCACGCAGACATCAAGACG-3′ (forward primer) for VEGFR-3; 5′-AGCTGCCTGACCACGCAATGT-3′ (reverse primer) and 5′-TTCCACGTGACCAGGGGTCCT-3′ (forward primer) for VEGFR-2; and 5′-GGATTCTGACTTAGAGGCGTTCAGT-3′ (reverse primer) and 5′-GTTCACCCACTAATAGGGAACGTGA-3′ (forward primer) for 28S (internal control).

### Western blotting

Cells were lysed with RIPA buffer (50 mM Tris, pH 7.4; 150 mM NaCl; 1% IGEPAL-CA 630 (Sigma-Aldrich); 1% Triton X-100; 1% sodium deoxycholate (Sigma-Aldrich); and 0.1% SDS) or cell lysis buffer 1× (9803, Cell Signaling) containing phosphatase and protease inhibitors (Complete and phosSTOP, Roche). Proteins were separated on acrylamide gels (10%) and transferred onto PVDF membranes. Proteins were detected by overnight incubation at 4 °C with the indicated antibodies followed by 1 h incubation at room temperature with horseradish peroxidase-coupled secondary antibody (7074 and 7076, Cell Signaling) and enhanced chemiluminescent substrate (NEL104001EA, PerkinElmer) using a LAS4000 imager (Fujifilm). The following antibodies were used: uPARAP (1/2500; mAb 2H9F12)^51^, pVEGFR-3 (Tyr1230/31) (1/1000; CY1115, Cell Application), VEGFR-3 (1/1000; MAB 3757, Millipore), pVEGFR-2 (Tyr1175, 2478), VEGFR-2 (2479), pCrkII (Y221, 3491), CrkII (3492), pAkt (Ser473, 9271), Akt (9272), pJNK (Thr183/Tyr185, 9251), JNK (9252), pERK1/2 (Thr202/Tyr204, 9101), ERK (9102) (All from Cell Signaling; 1/1000), pPaxillin (Ser178) (1/1000; PP1051, BD Biosciences), pPaxillin (Tyr118) (1/1000; 2541, Cell Signaling), Paxillin (1/1000; AB 3794, Millipore), and GAPDH (1/10,000; MAB 374, Millipore). When analysing phosphorylated and total forms of a protein, blots were stripped and reprobed. Blots were run on parallel gels when proteins of the same molecular weigth were analysed. Quantifications were performed using Quantity One software. Full blots are shown in Supplementary Fig. 7–21.

### Pull-down assay for Cdc42 and Rac1

The assay was performed as previously described^52^. Briefly, cells were chilled on ice and lysed in ice-cold buffer containing 0.5% Triton X-100, 25 mM HEPES (pH 7.3), 150 mM NaCl, 4% glycerol, 0.1 mM AEBSF, 4 µg/ml aprotinin and 5 mM DTT. Lysates were centrifuged for 6 min at 16,000×*g*. Supernatants were immediately frozen in liquid nitrogen and stored at –80 °C until use. An aliquot of each supernatant collected before freezing was denatured in SDS-PAGE lysis buffer to measure the total Rac1 and Cdc42 content by Western blotting with mouse monoclonal antibodies (from Millipore (1/2000; 05-389) and BD Biosciences (1/500; 610929), respectively). For pull-down assays, supernatants were incubated for 30 min at 4 °C with 30 µg of GST-PBD protein containing the Cdc42-binding and Rac-binding region of PAK-1B linked to glutathione-Sepharose beads. The beads were washed four times in lysis buffer and boiled in 60 µl of SDS-PAGE lysis buffer. For loading control, samples were incubated for 3 h at RT with a mouse anti-human GAPDH antibody (1/2000; Millipore).

### Cell proliferation assay

Cellular growth was measured using the CyQUANT cell proliferation assay kit following manufacturer’s instructions (C7026, Invitrogen). LECs (2 × 10^4^) were seeded in a 96-well plate and allowed to attach for 8 h. After washing, cells were cultured in complete medium (EGM-2) or in EBM-2 supplemented with 1% FBS with or without recombinant factors (400 ng/ml rhVEGFC or 10 ng/ml rhVEGF-A). After 48 h, medium was discarded, and plates were frozen at −80 °C. After thawing the plates the day of the assay, cells were lysed, and total cellular nucleic acid was measured using fluorescence at 520 nm emission after excitation at 480 nm.

### Scratch assay

LECs (2 × 10^5^) were seeded in a 24-well plate and allowed to attach for 12 h. Scratches were made with a 10 µl tip. Cells were rinsed with PBS and cultured in EBM-2 supplemented with 1% FBS and mitomycin C (0.1 µg/ml; M4287, Sigma-Aldrich). Recombinant factors were either added or not to the culture medium. Images were captured after 17 h with an epifluorescence microscope (Nikon Eclipse Ti, 60× magnification). To obtain the percentage of scratch opening, the area unoccupied by LECs at *T* = 17 was divided by the area unoccupied by LECs at *T* = 0. The percentage of scratch closure corresponds to subtraction of the percentage of scratch opening from 100%.

### Migration assay

Sterile 8-µm pore polycarbonate filters (3422, Corning) were coated overnight with 100 µl of gelatin (0.2%). The lower compartment of a 24-well plate was filled with 600 µl of EBM-2 containing 1% FBS and VEGF-A (10 ng/ml), VEGF-C (400 ng/ml) or VEGF-C_Cys156Ser_ (500 ng/ml). LECs (5 × 10^4^) were seeded in the upper compartment in 300 µl of EBM-2 supplemented with 0.5% FBS. After 24 h incubation at 37 °C in a humidified atmosphere (5% CO_2_), cells were fixed with methanol for 30 min at −20 °C and stained with Giemsa Azure Eosin Methylene Blue solution (1.09204.0500, Millipore) diluted 1/25 in distilled water. The filters were removed, and cells on the upper side of the filter were gently removed with a cotton swab. Cells were counted in at least six separate fields under a light microscope (AH3-RFCA, Olympus) at 40× magnification.

### Directional migration assay

LECs (2 × 10^4^) in EGM-2 MV complete medium were allowed to attach for 4 h in a µ-slide chemotaxis 2D assay (80326, Ibidi). After washing, cells were maintained in serum-free medium, and chamber chemoattractant was added (10 ng of rhVEGF-A, 400 ng of rhVEGFC or 20% complete medium) to the upper chamber. LECs migration was observed for 20 h with a Nikon A1R confocal microscope at 10X magnification. Analyses were performed using Ibidi software (Ibidi)^53^.

### Immunofluorescence

For phalloidin and uPARAP stainings, LECs were fixed in 1% paraformaldehyde in PBS at room temperature for 10 min and then blocked with 10% NGS, 5% BSA and 0.2% saponin for 1 h at room temperature. Cells were then incubated with uPARAP antibody (1/100; mAb 2H9F12)^51^ overnight at 4 °C. After washing, phalloidin-Atto550 (1/500; 19083, Sigma-Aldrich) was added with goat anti-mouse secondary antibody (1/200; AF488) for 1.5 h at room temperature. Vectashield mounting medium with DAPI was used to mount µ-slide VI (Ibidi). Image acquisition was performed on an epifluorescence microscope (Nikon Eclipse Ti, 60× magnification).

### Proximity ligation assay of protein-protein interactions

After different time periods, LECs were fixed in 4% paraformaldehyde for 20 min at room temperature and washed three times with PBS. After washing, cells were blocked and permeabilised for 1 h at room temperature with PBS containing 2% BSA, 10% NGS, and 0.3% saponin. Cells were then incubated with mouse anti-uPARAP (1/100; mAb 2H9F12^51^), rabbit anti-VEGFR-2 (1/100; 2479, Cell Signaling), mouse anti-VEGFR-3 (1/100; MAB 3757, Millipore), rabbit anti-VEGFR-3 (1/100; 20R-2485, Fitzgerald) and mouse anti-Flag (1/100; F1804, Sigma-Aldrich) antibodies diluted in blocking buffer. Proximity ligation was performed according to the manufacturer’s protocol using the Duolink Detection Kit with PLA PLUS and MINUS Probes for mouse and rabbit (DUO92002, DUO92004, Sigma-Aldrich). Negatives controls correspond to cells only incubated with one of the two primary antibodies. Vectashield mounting medium with DAPI was used to mount coverslips. Image acquisition was performed using an epifluorescence microscope (Nikon Eclipse Ti, 60× magnification), and automatic quantifications were performed by a computer-assisted method measuring the number of dots per cell. Image processing and measurements were performed using the image analysis toolbox of Matlab R2016a (9.0.341360; Matworks, Inc., Natick, MA, USA). PLA experiments were also performed on migrating LECs. LECs (1 × 10^4^) were seeded in a µslides VI^0.4^ (80606, Ibidi) and chemoattractant (400 ng of rhVEGF-C) was added in the upper chamber. After 6 h of migration towards the VEGF-C gradient, cells were fixed and PLA was performed as described.

### Immunoprecipitation

uPARAP was isolated by binding to an antibody directed against uPARAP (mouse monoclonal uPARAP (1/100; mAb 2H9F12) overnight at 4° in Cell Lysis Buffer. As a negative control, immunoglobulin G (IgG) was used instead of the specific antibody during immunoprecipitation. After a 4 h precipitation using Dynabeads Protein G (10004D, ThermoFisher), proteins were released from the beads by heating for 5 min at 95 °C in Cell Lysis Buffer and were subjected to Western blotting.

### Statistics

Results were analysed with GraphPad Prism 5.0 and were expressed as means ± SEM of different experiments. *P* values were obtained using the Mann-Whitney test, two-tailed (not assuming Gaussian distributions), or Chi-square test, and are shown in figures as *P < 0.05, **P < 0.01, ***P < 0.001, ****P < 0.0001. *P* < 0.05 was considered as statistically significant. The experiments were not randomised. No blinding was done in the analysis and quantifications.

## Electronic supplementary material

Supplementary Information

Reporting Summary

## Data Availability

All relevant data are available from the corresponding author upon request. A reporting summary for this Article is available as a Supplementary Information file.
