# Supplementary material for: uPARAP/Endo180 receptor is a gatekeeper of VEGFR-2/VEGFR-3 heterodimerisation during pathological lymphangiogenesis
Source: Nat Commun. 2018 Dec 5;9:5178. doi: 10.1038/s41467-018-07514-1 (PMC6281649; doi:10.1038/s41467-018-07514-1)
Supplement: Supplementary file 1 — Supplementary Information [file 41467_2018_7514_MOESM1_ESM.pdf]

Supplementary Information

uPARAP/Endo180 receptor is a gatekeeper of VEGFR-2/VEGFR-3 heterodimerisation during pathological lymphangiogenesis

Durré et al.

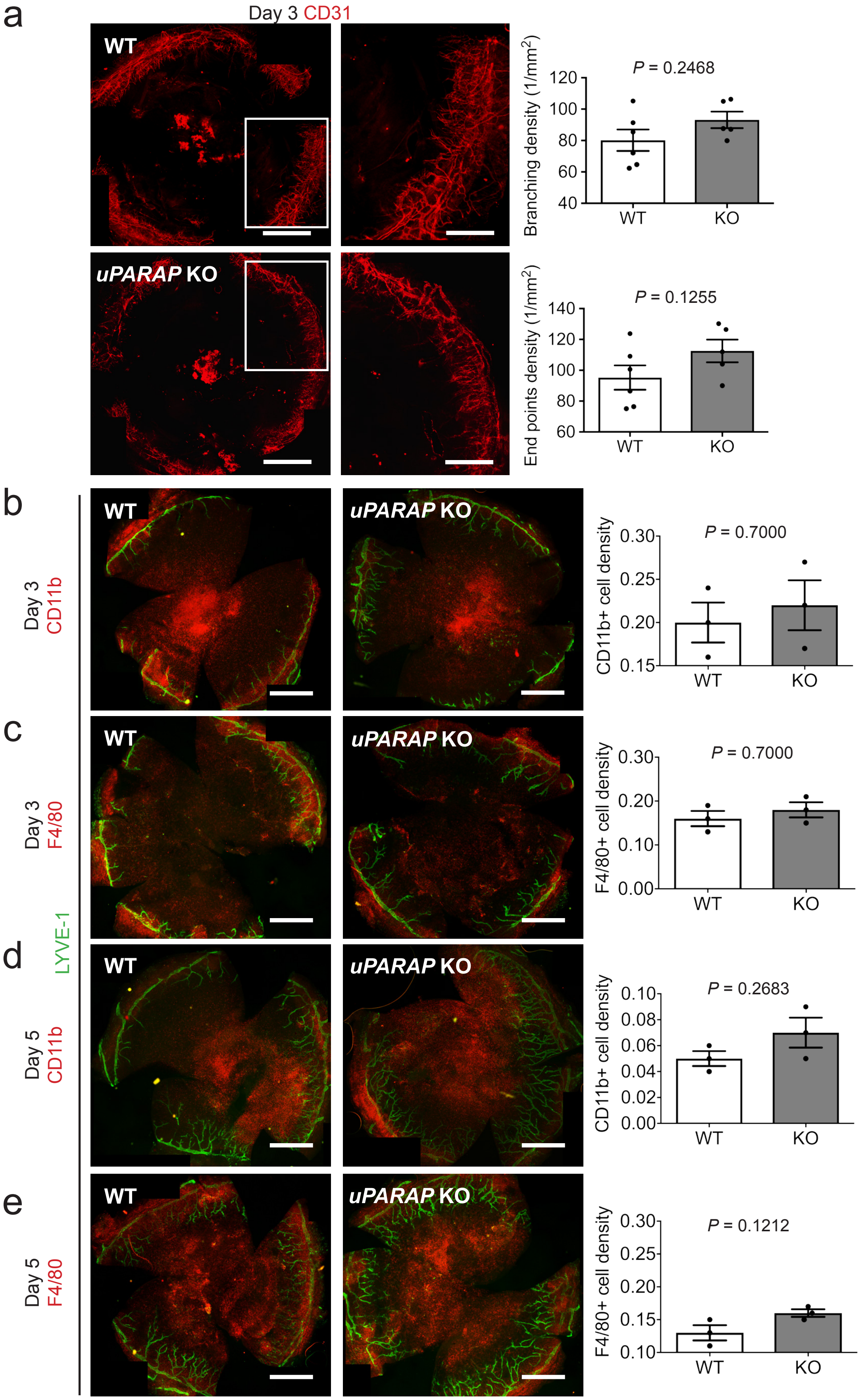

Supplementary Figure 1. Corneal angiogenesis and inflammatory cell infiltration are not affected by uPARAP status. Whole-mounted cornea at day 3 or day 5 (as indicated) after thermal cauterization induced in *uPARAP* KO and WT mice. (a) Blood vessel visualization (n = 6 for WT and n = 5 for KO mice). Bars = 1 mm and 500 µm in left and right (higher magnification of the insert) images, respectively. Histograms correspond to a computerized quantification of the branching and end points density. (b-e) Double immunostaining of LYVE-1 (green) with CD11b (red) (b, d) or F4/80 (red) (c, e). Histograms represent the quantification of inflammatory cell infiltration in the corneas (n = 3) conducted according to Detry et al.[1]. Bars = 1 mm. All results are expressed as mean ± SEM. Statistical analyses were performed using a non-parametric Mann-Whitney test.

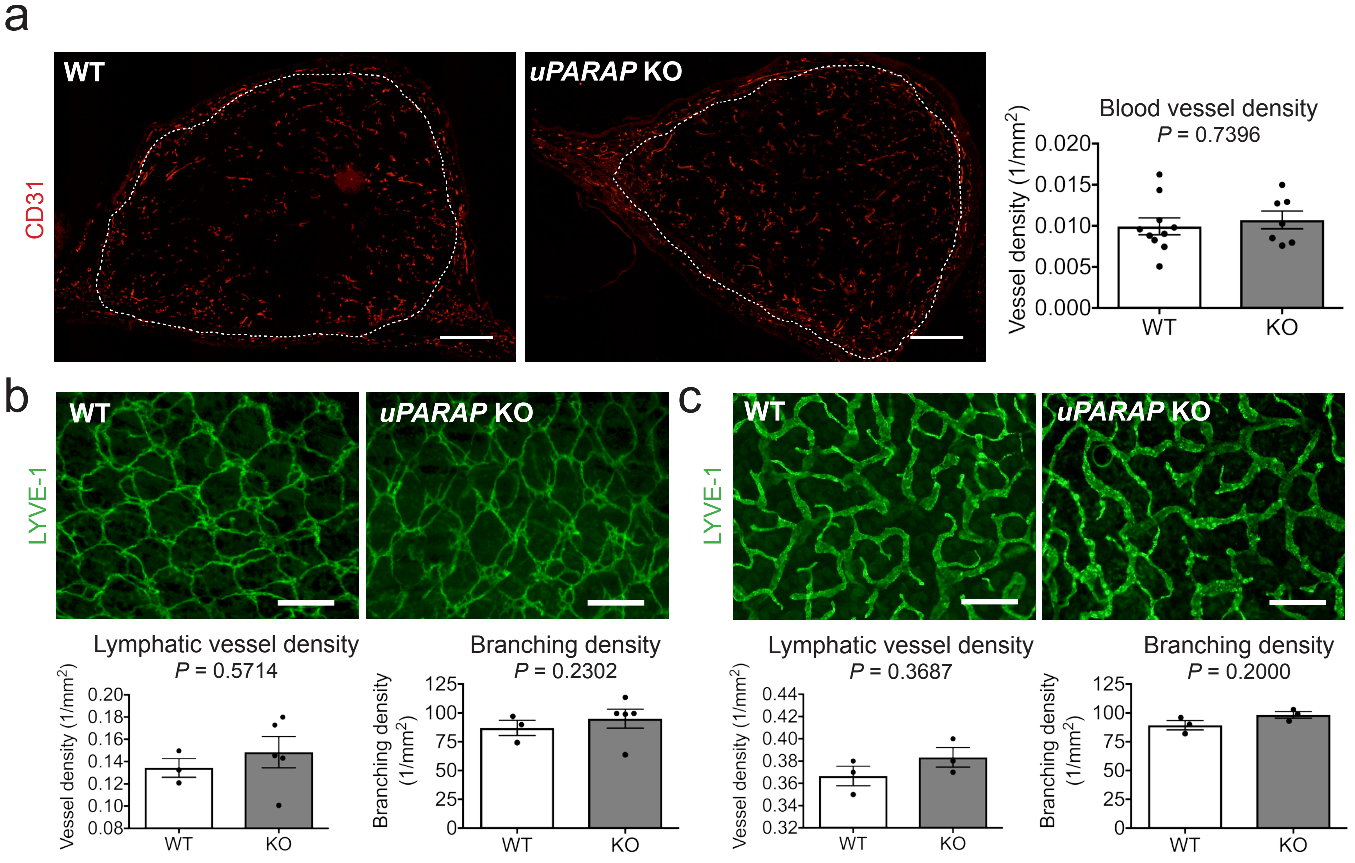

Supplementary Figure 2. uPARAP deficiency does not affect tumoral angiogenesis and lymphatic vasculature formation under physiological conditions. (a) Ear sponge assay using gelatin sponges soaked with PyMT tumor cells implanted in ears of WT mice or *uPARAP*-deficient (*uPARAP* KO) mice. Blood vessels were analyzed by CD31 (red) immunostaining. White dots delineate the sponge in the ear. Histograms represent the area density of vessels quantified by a computer-assisted method (n = 10 for WT and n = 7 for KO mice). Bars = 500 µm. (b) Dermal lymphatic vasculature on whole-mounted post-natal tail (P6) (n = 3 for WT and n = 5 for KO mice) (b) and adult ears (n=3) (c) from *uPARAP* (KO)-deficient mice and WT mice. Bars = 1 mm. Histograms represent the computerized quantifications of lymphatic vasculature parameters conducted according to Detry et al.[2]. All results are expressed as mean ± SEM, and statistical analyses were performed using a non-parametric Mann-Whitney test.

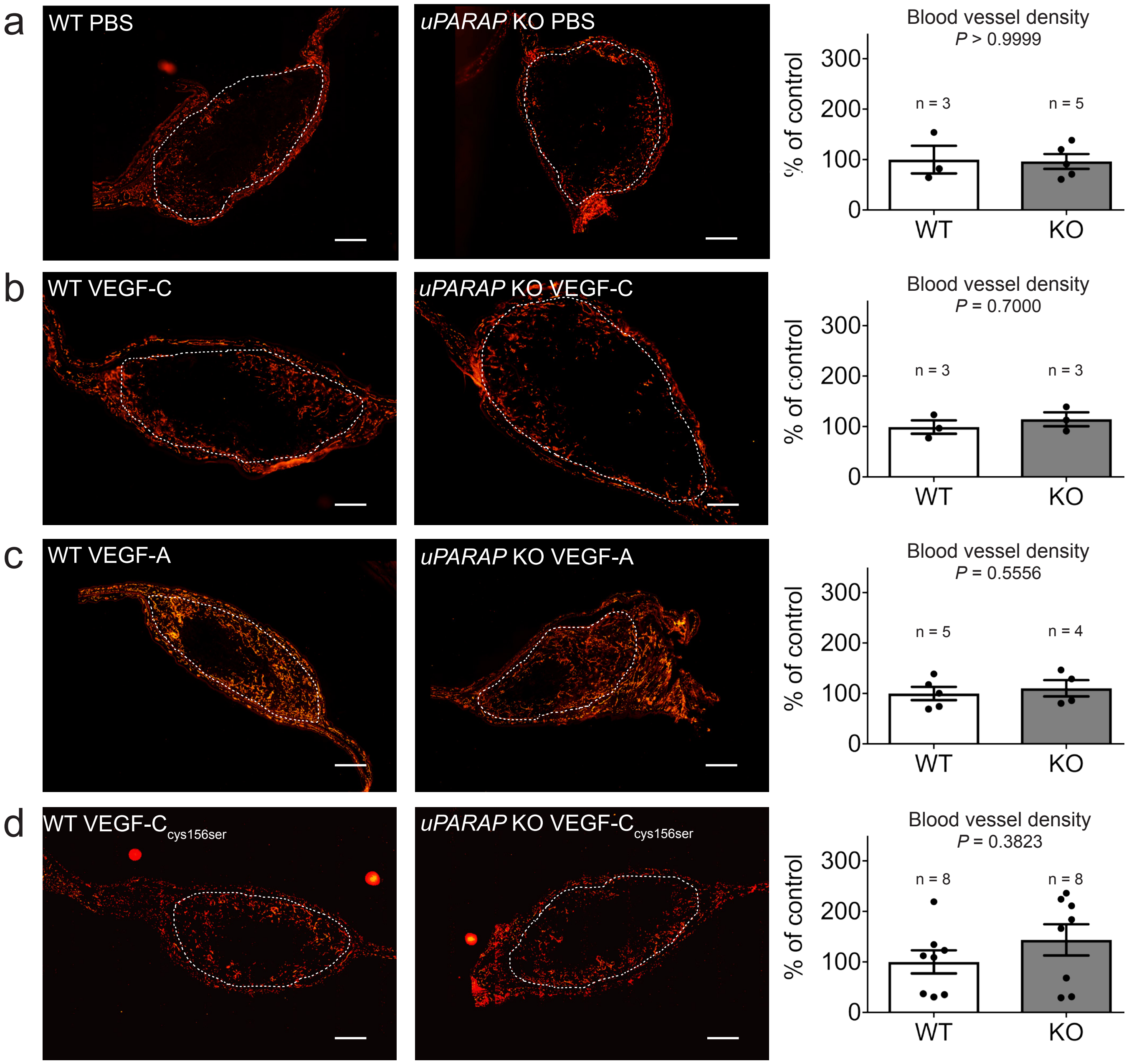

Supplementary Figure 3. uPARAP deficiency does not affect angiogenesis. Gelatin sponges soaked with PBS (a), VEGF-C (b), VEGF-A (c) or mutated VEGF-$C_{Cys156Ser}$ (d) were implanted in mouse ears. White dots delineate the sponge in the ear. Blood vasculature was examined by CD31 (red) immunostaining. Histograms represent the area density of vessels quantified by a computer-assisted method and expressed as percentage of WT control (n = 3-8 as indicated). Bars = 500 µm. All results are expressed as mean ± SEM, and statistical analyses were performed using a non-parametric Mann-Whitney test.

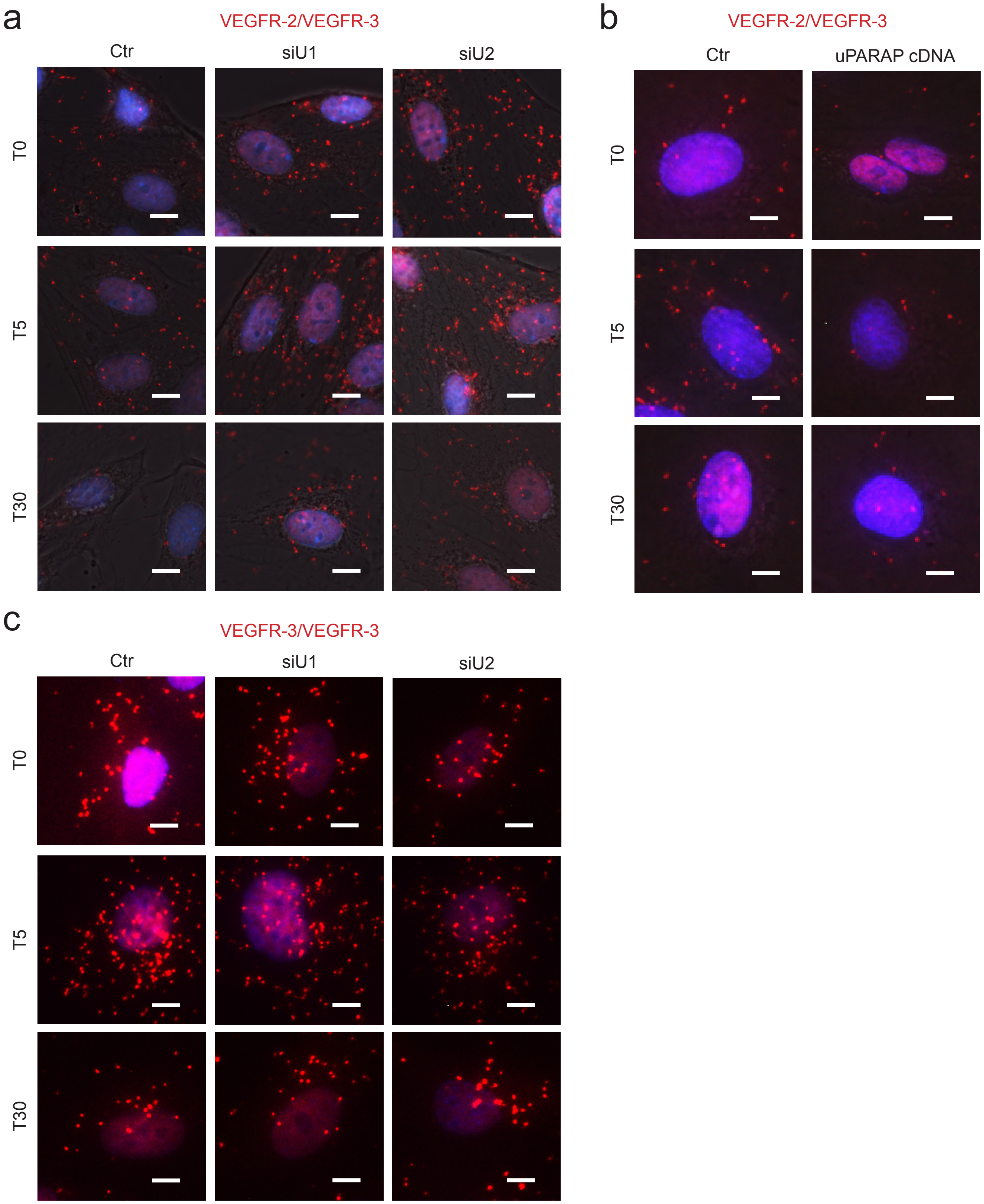

Supplementary Figure 4. uPARAP restricts VEGFR-2/VEGFR-3 heterodimerisation. Representative images of in situ PLA detection of VEGFR-2/VEGFR-3 heterodimerisation (a, b) in control LECs (Ctr) (a, b), uPARAP-deficient LECs (siU1 and siU2) (a), LECs overexpressing uPARAP (uPARAP cDNA) (b). (c) Representative images of in situ PLA detection of VEGFR-3/VEGFR-3 homodimerisation in control LECs (Ctr) and uPARAP-deficient LECs (siU1 and siU2). Cells were stimulated or not with VEGF-C for 5 or 30 minutes, and protein-protein interactions are indicated by red dots. Bars = 10 µm.

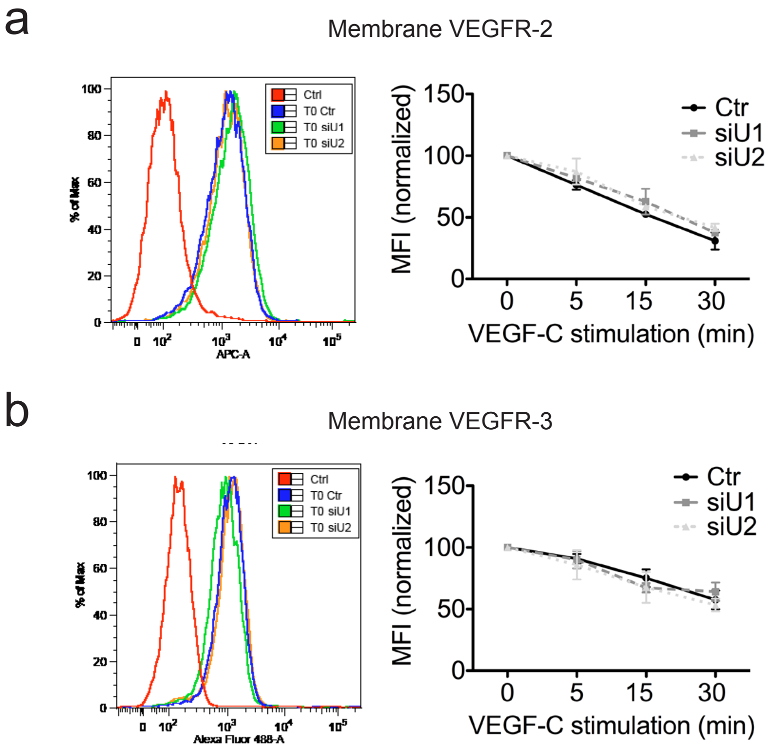

Supplementary Figure 5. uPARAP silencing does not influence VEGFR-2 or VEGFR-3 internalisation. Flow cytometry analysis of cell surface VEGFR-2 (a) and VEGFR-3 (b) in non-permeabilized LECs transfected (siU1 and siU2) or not (Ctr) with siRNA. In basal condition (at T0), median fluorescence intensity (MFI) was similar in Ctr and siU-treated LECs (left panels). After VEGF-C stimulation, the kinetics of VEGF receptor disappearance at the cell surface were similar in Ctr and silenced LECs (right panels). Results are expressed as the % of receptor amount detected in basal condition (T0) (n = 3, biological triplicates).

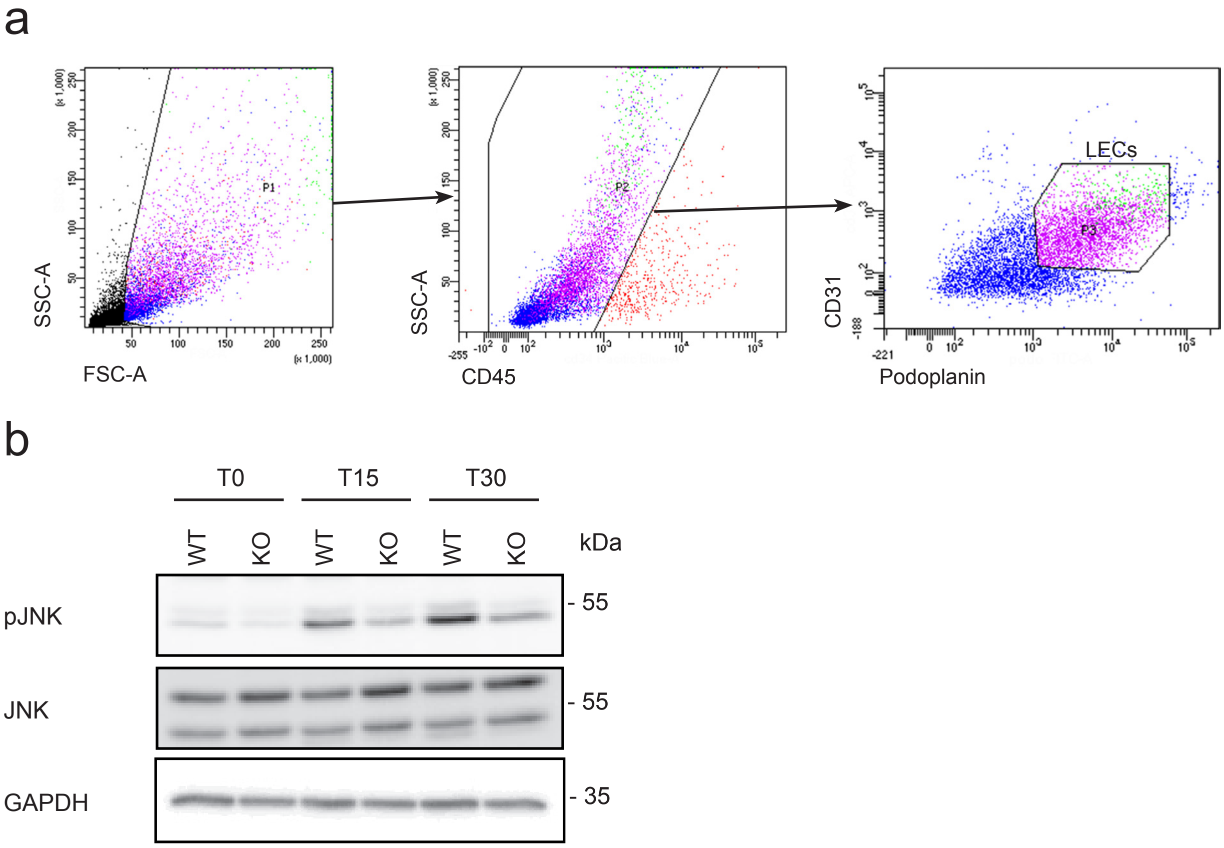

Supplementary Figure 6. uPARAP down-regulation impairs JNK phosphorylation in LECs isolated from *uPARAP* KO mice. (a) Gating strategies to sort LECs ($CD45^{-}CD31^{+}Podoplanin^{+}$) from *uPARAP* -/- and WT mice for in vitro cultures presented on Fig. 3a and Supplementary Fig. 6b. (b) LECs were isolated from lungs of *uPARAP* WT and KO mice and stimulated for the indicated time (minutes) with VEGF-C. Western blots of total (JNK) and phosphorylated (pJNK). GAPDH is used as loading control.

Fig. 3a

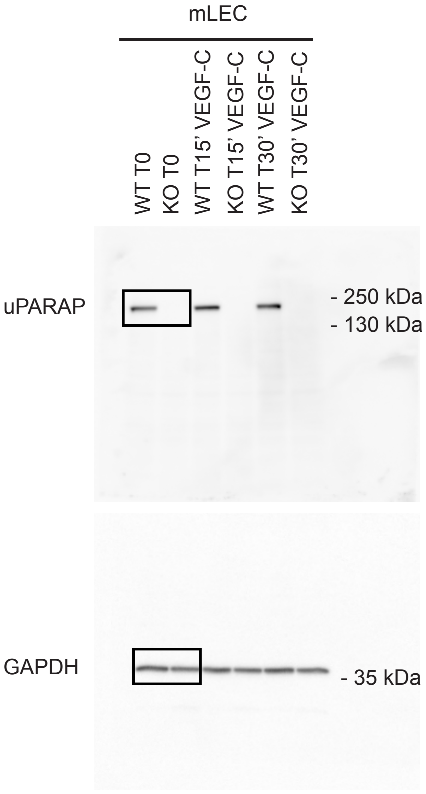

Fig. 3b

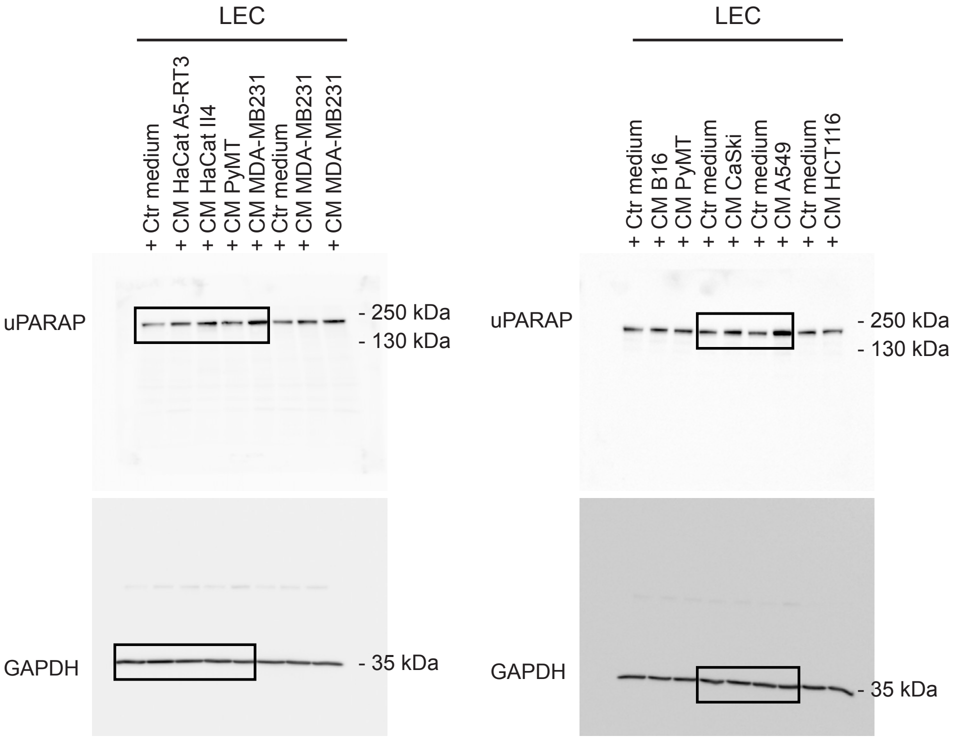

Supplementary Figure 7. Full Western blots. Portions of blots presented in Fig. 3a, b are indicated.

Fig. 4a

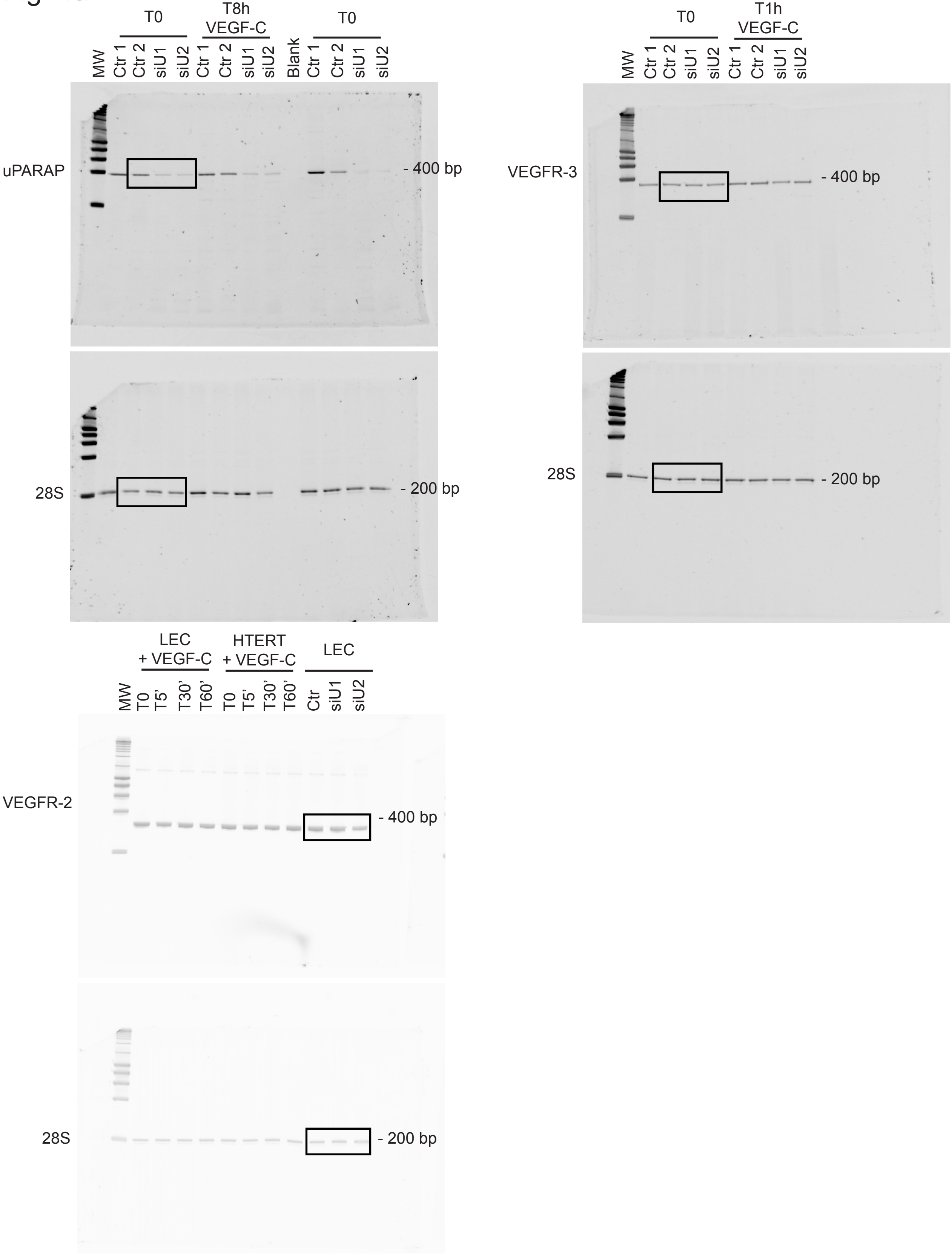

Supplementary Figure 8. Full RT-PCR product electrophoresis gels. Portions of gels presented in Fig. 4a are indicated.

Fig. 4b

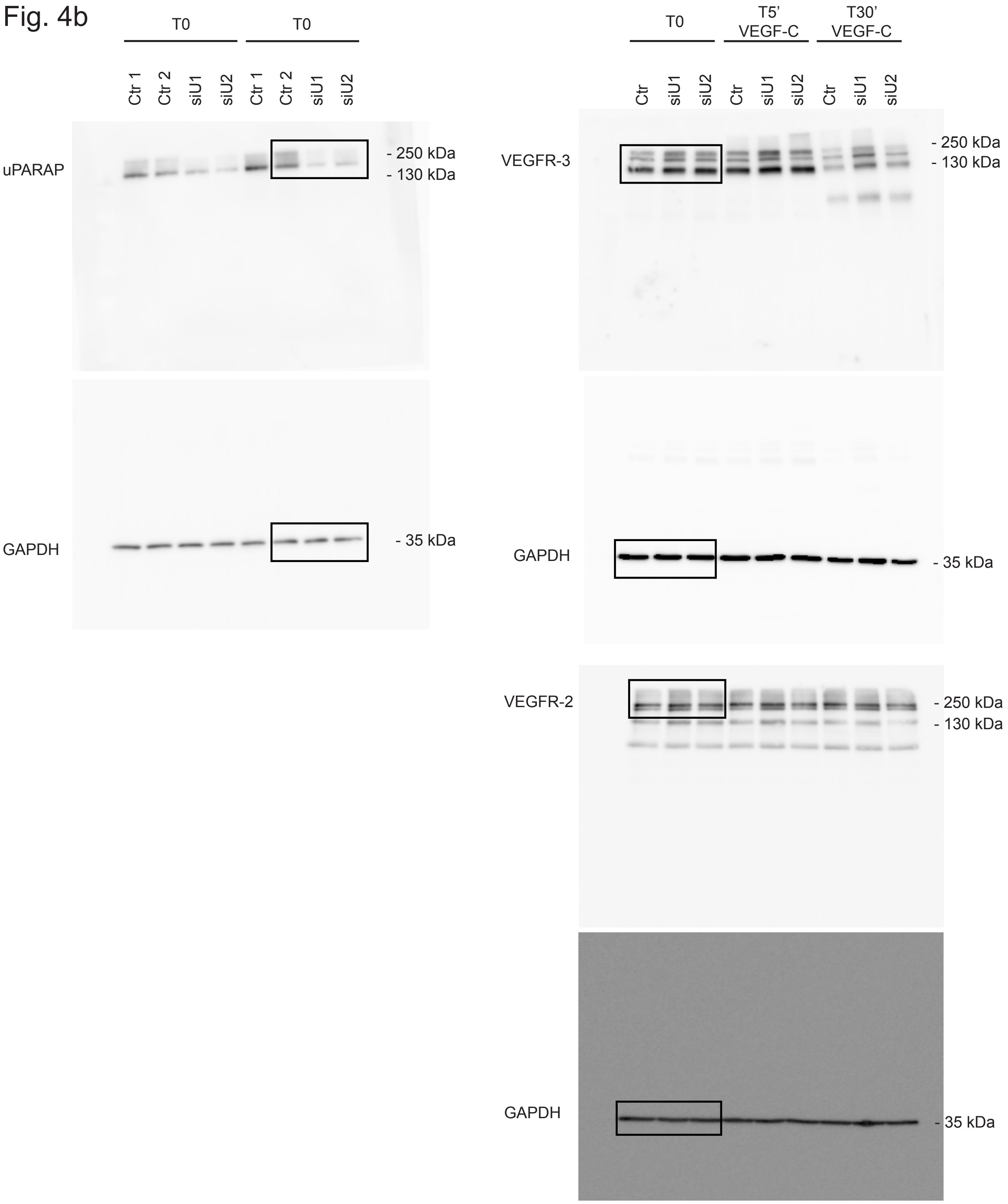

Supplementary Figure 9. Full Western blots. Portions of blots presented in Fig. 4b are indicated.

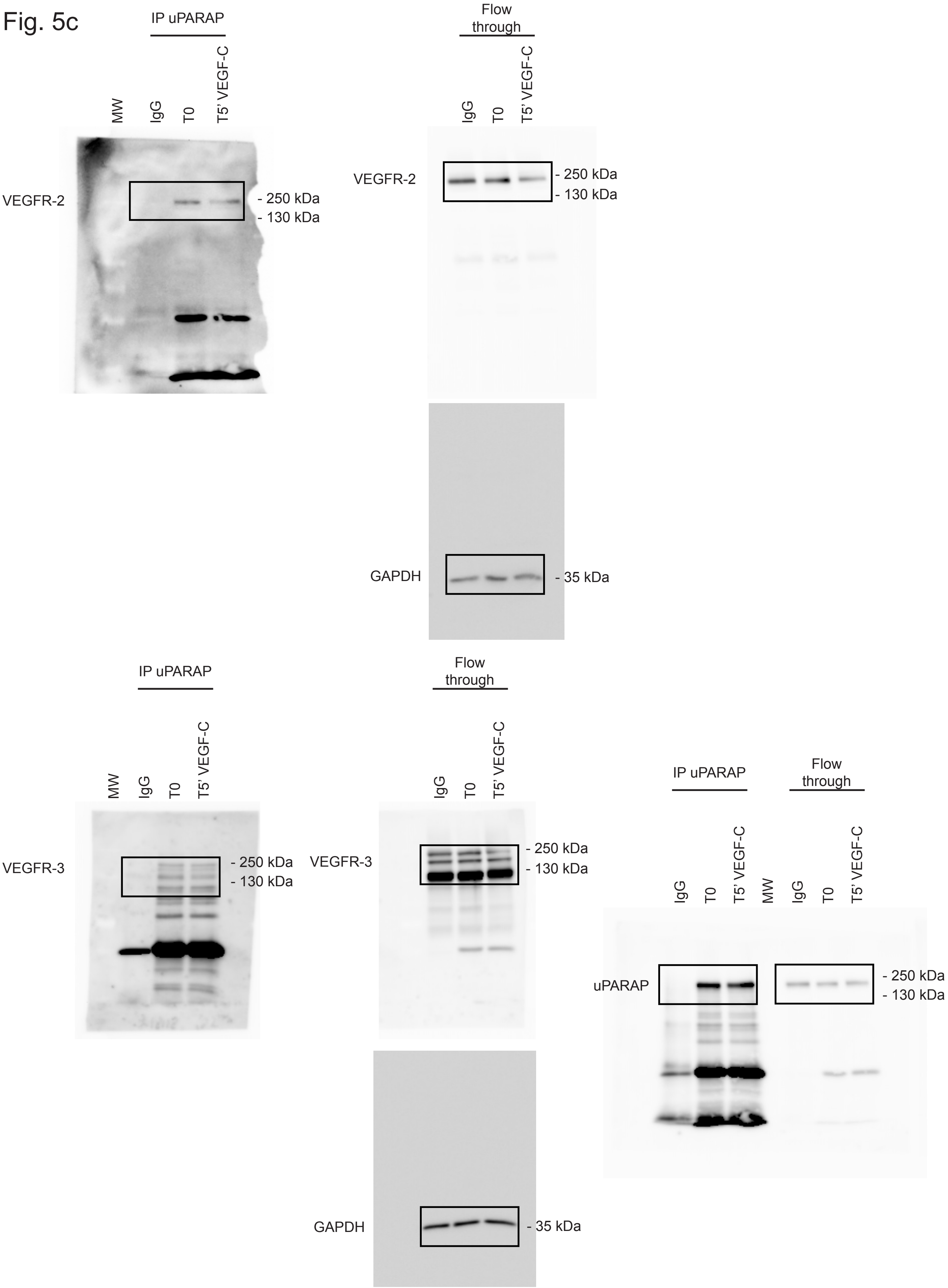

Supplementary Figure 10. Full Western blots. Portions of blots presented in Fig. 5c are indicated.

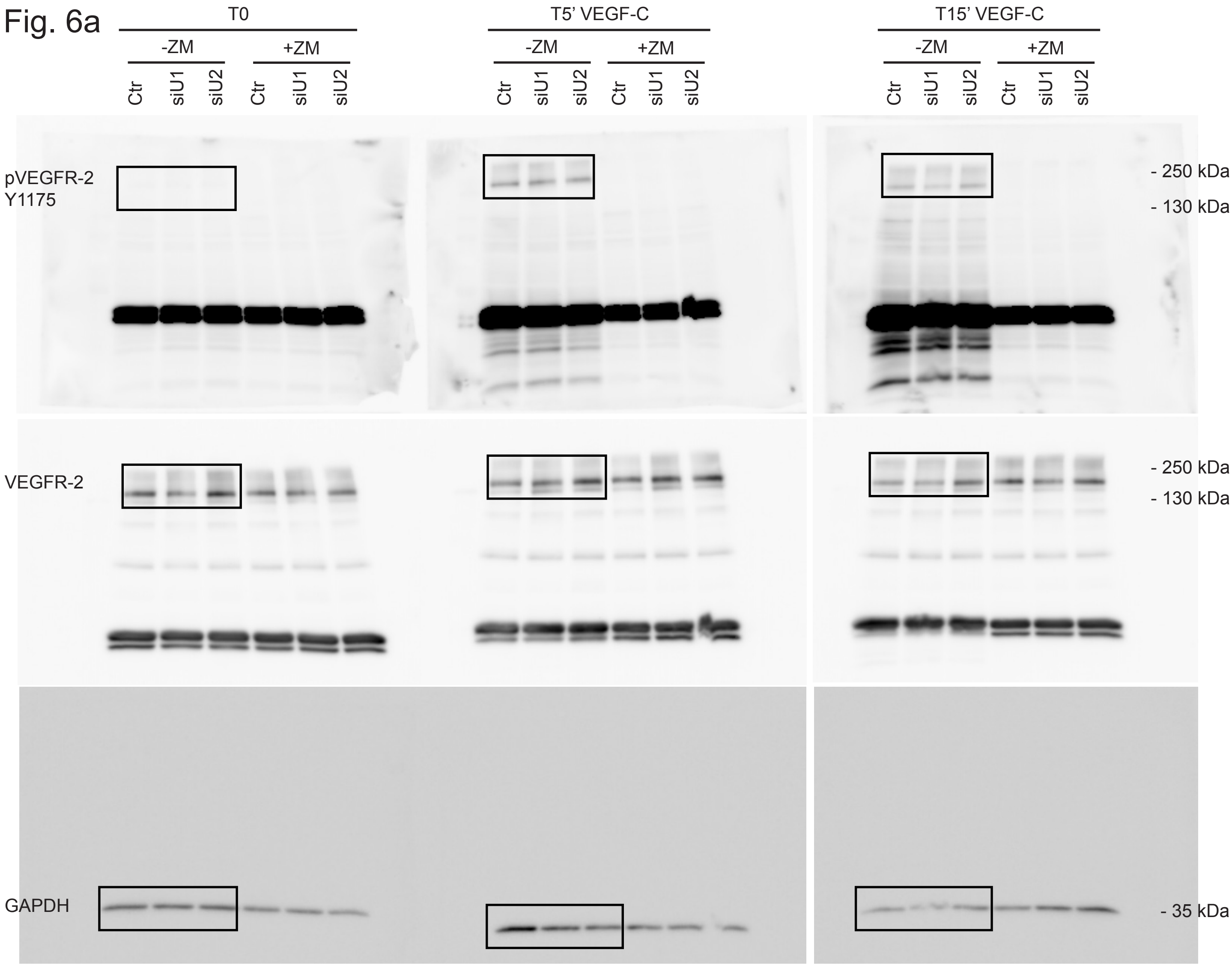

Supplementary Figure 11. Full Western blots. Portions of blots presented in Fig. 6a are indicated.

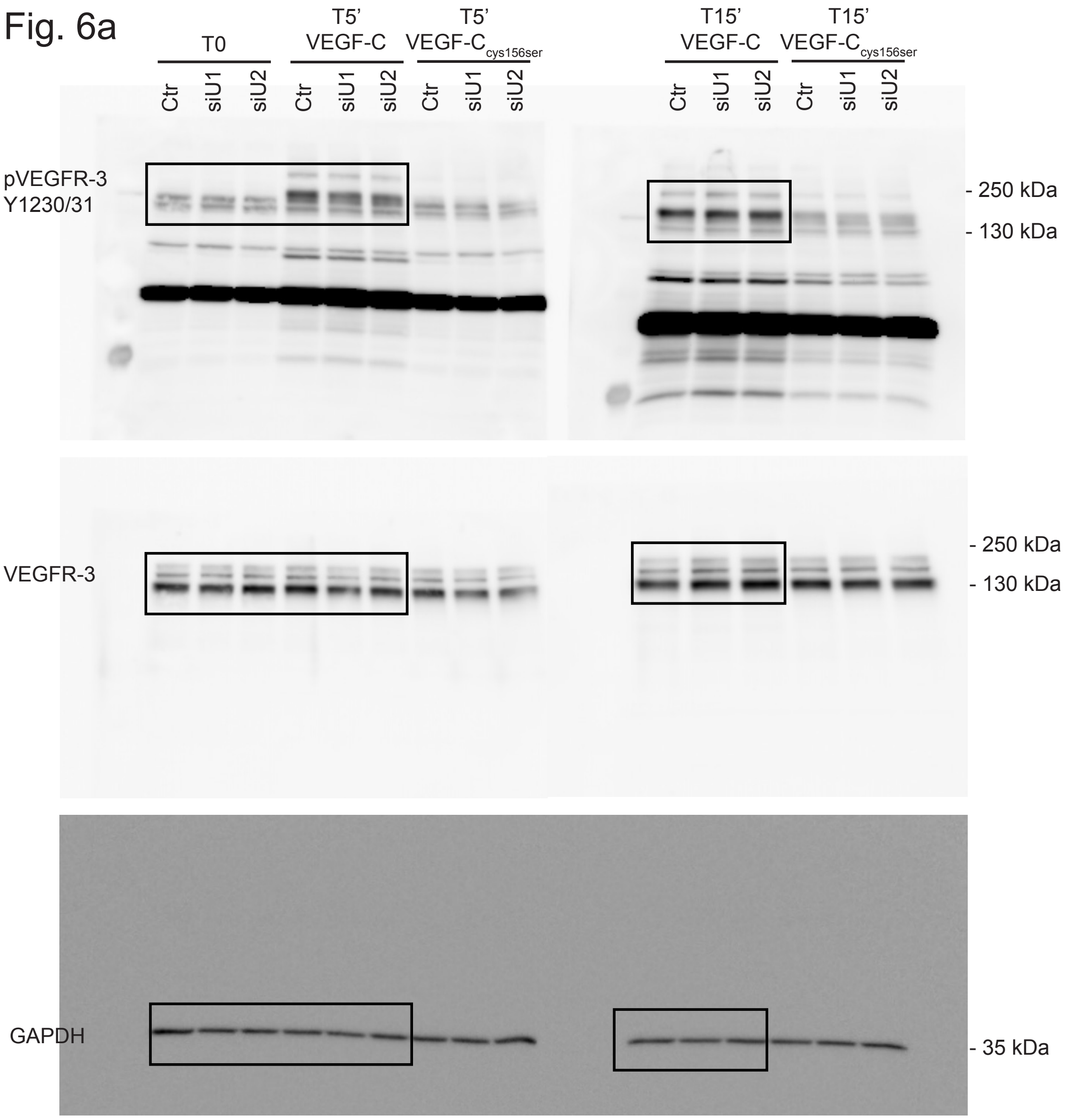

Supplementary Figure 12. Full Western blots. Portions of blots presented in Fig. 6a are indicated.

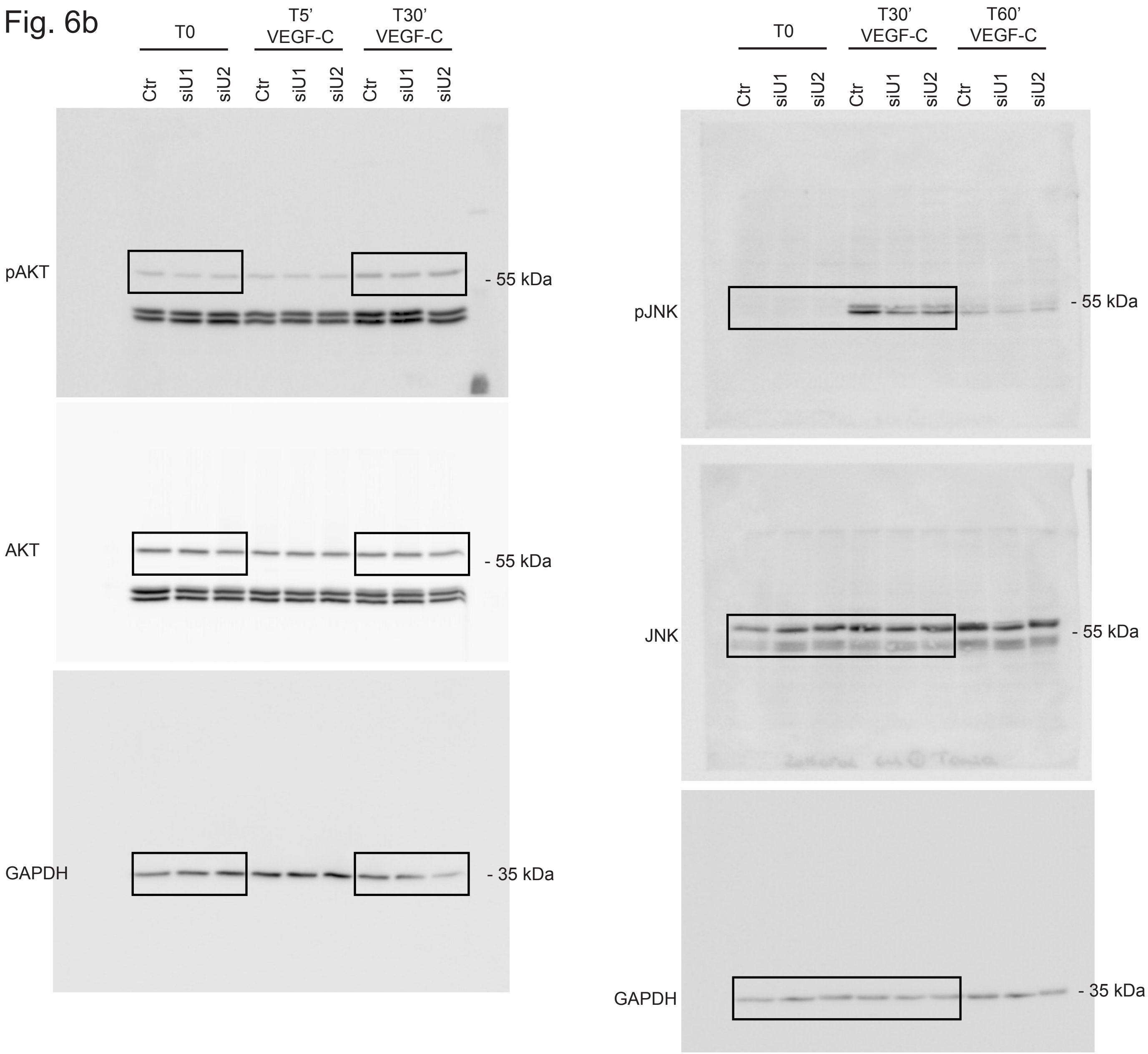

Supplementary Figure 13. Full Western blots. Portions of blots presented in Fig. 6b are indicated.

Fig. 6b

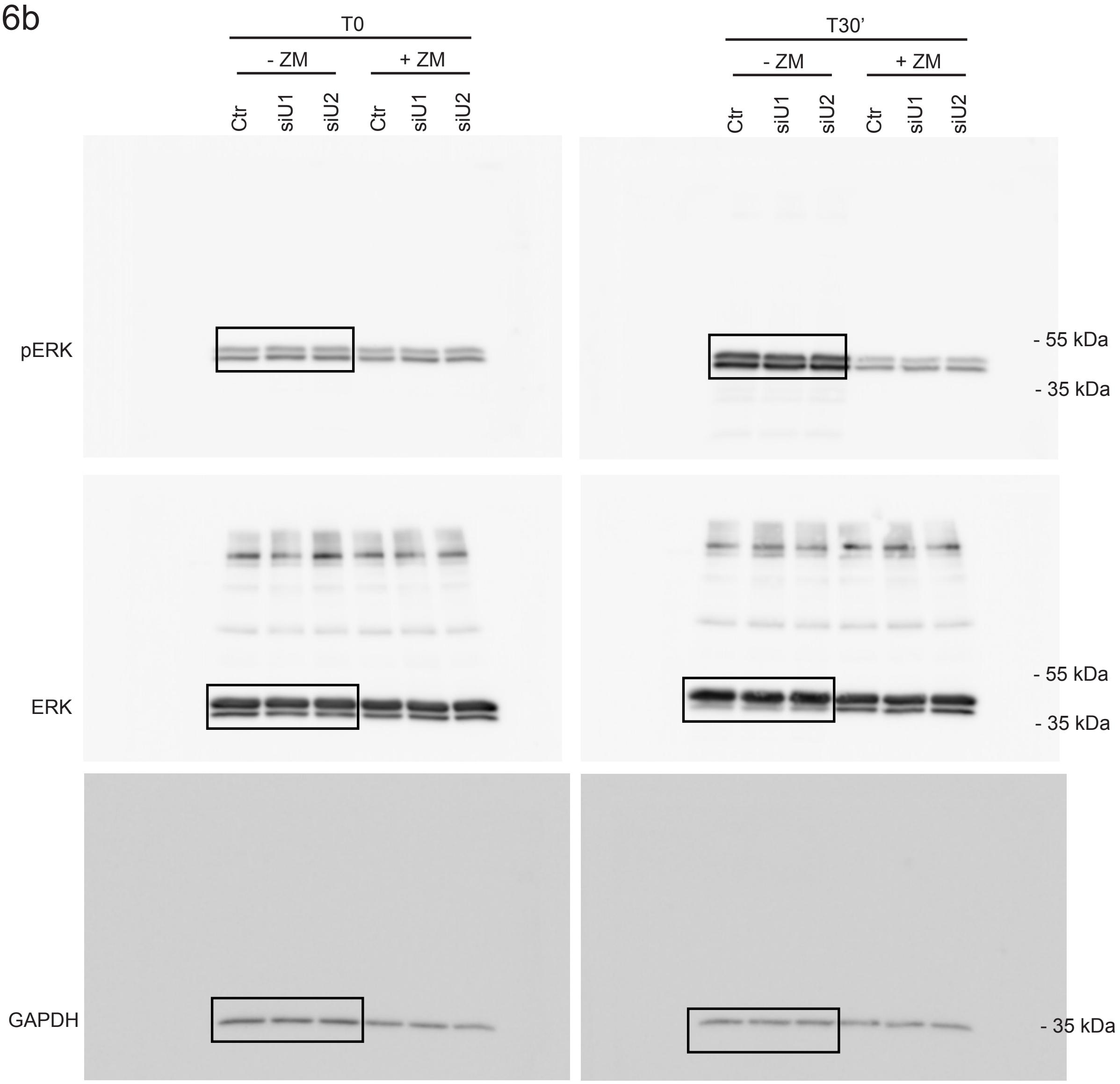

Supplementary Figure 14. Full Western blots. Portions of blots presented in Fig. 6b are indicated.

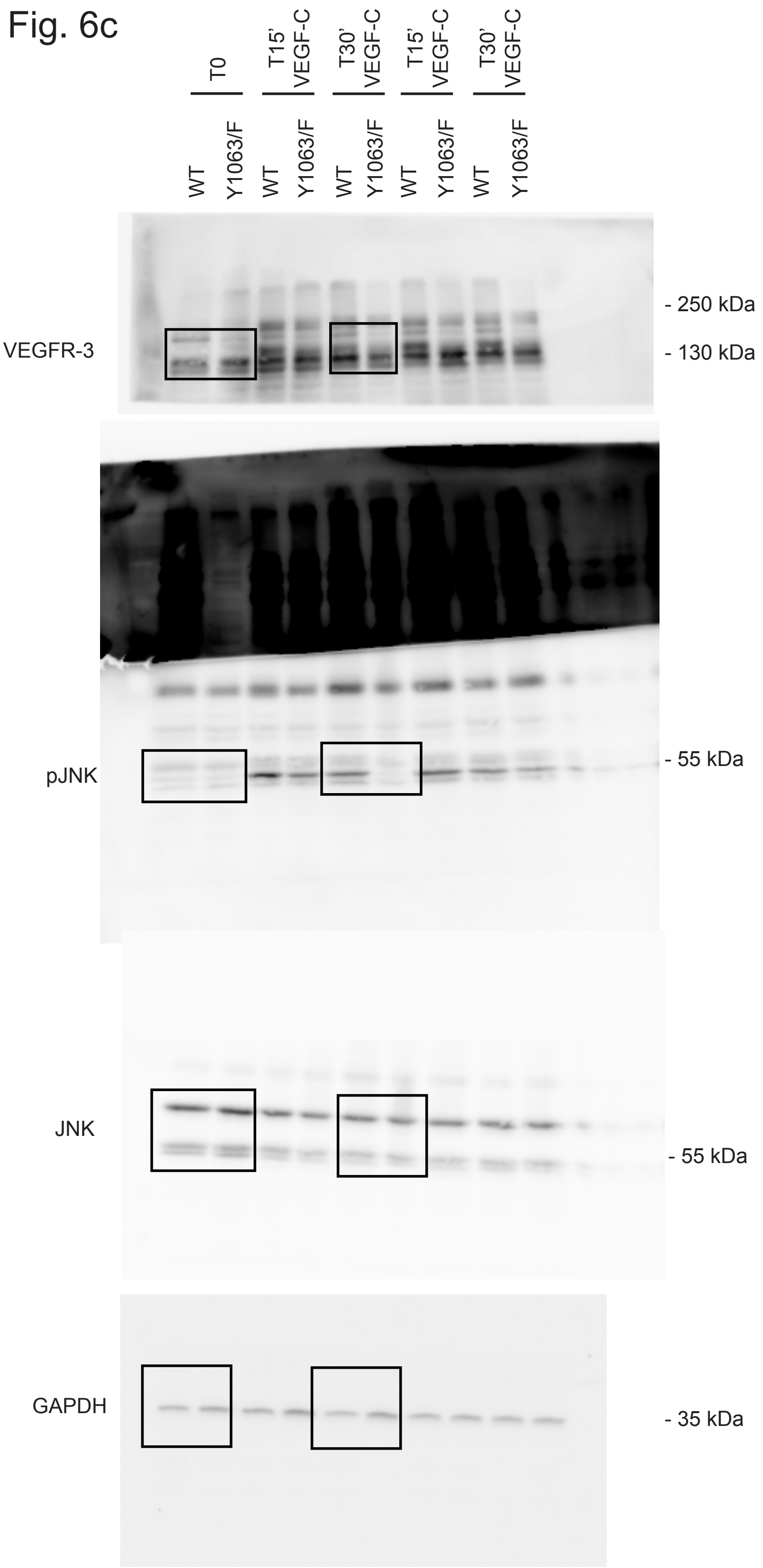

Supplementary Figure 15. Full Western blots. Portions of blots presented in Fig. 6c are indicated.

Fig. 6d

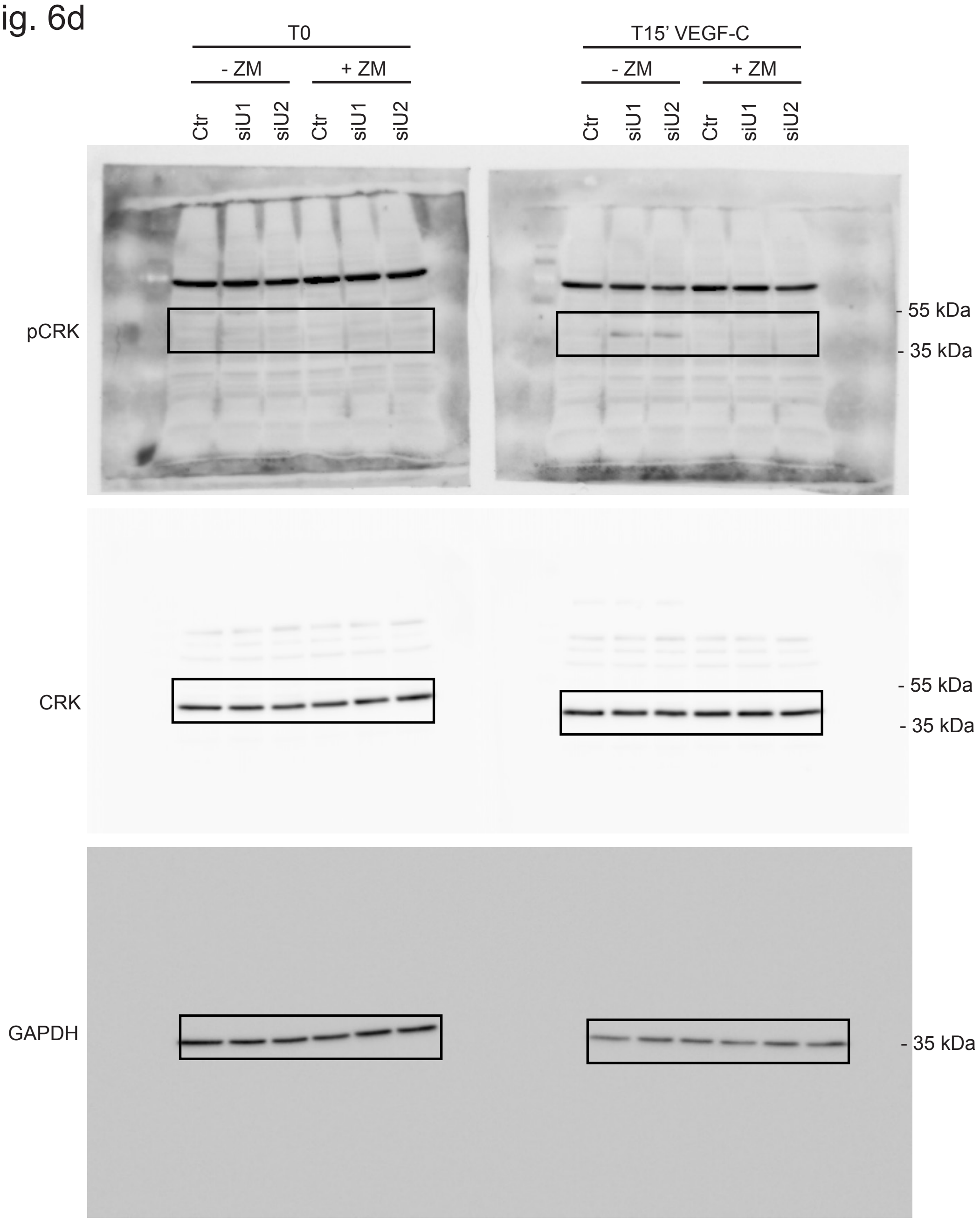

Supplementary Figure 16. Full Western blots. Portions of blots presented in Fig. 6d are indicated.

Fig. 6e

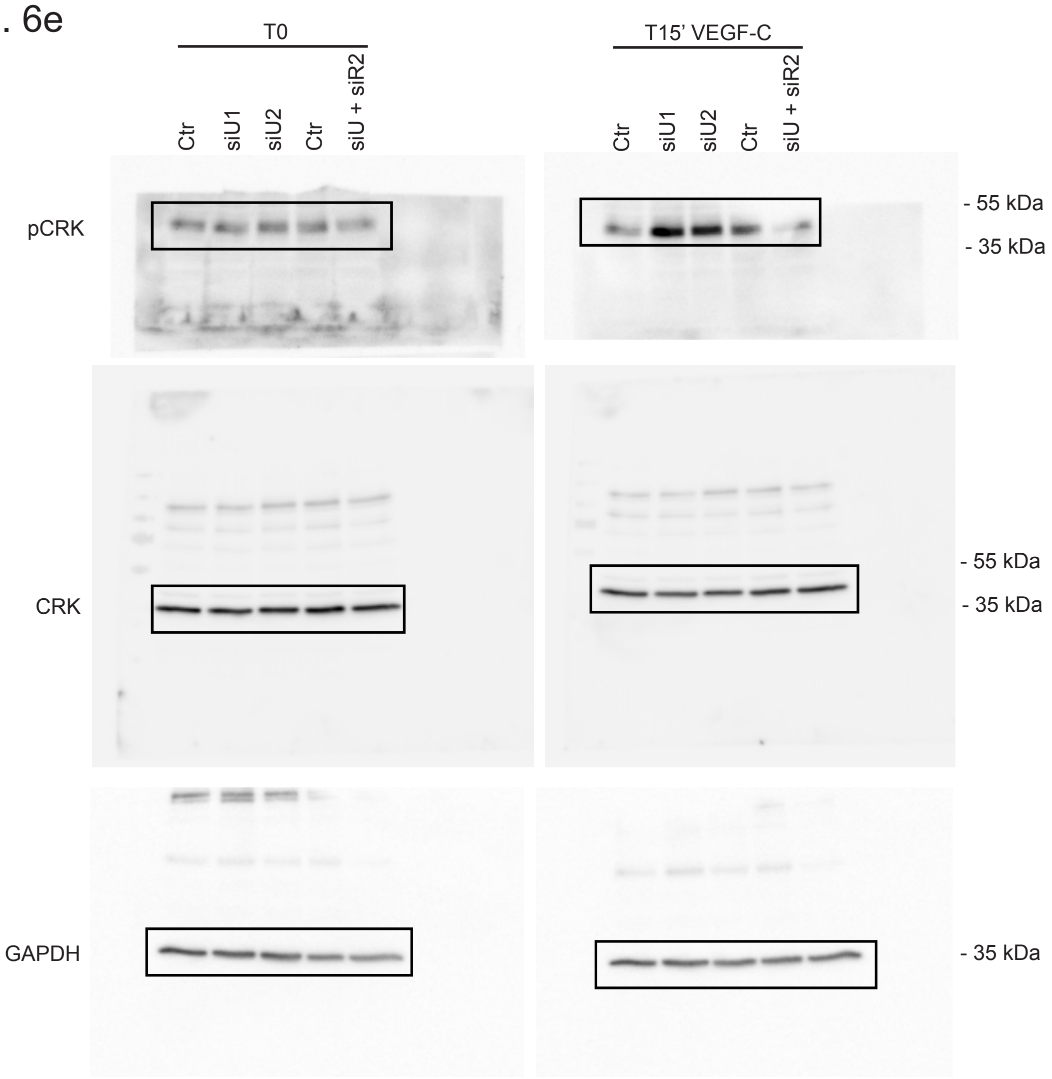

Supplementary Figure 17. Full Western blots. Portions of blots presented in Fig. 6e are indicated.

Fig. 6f

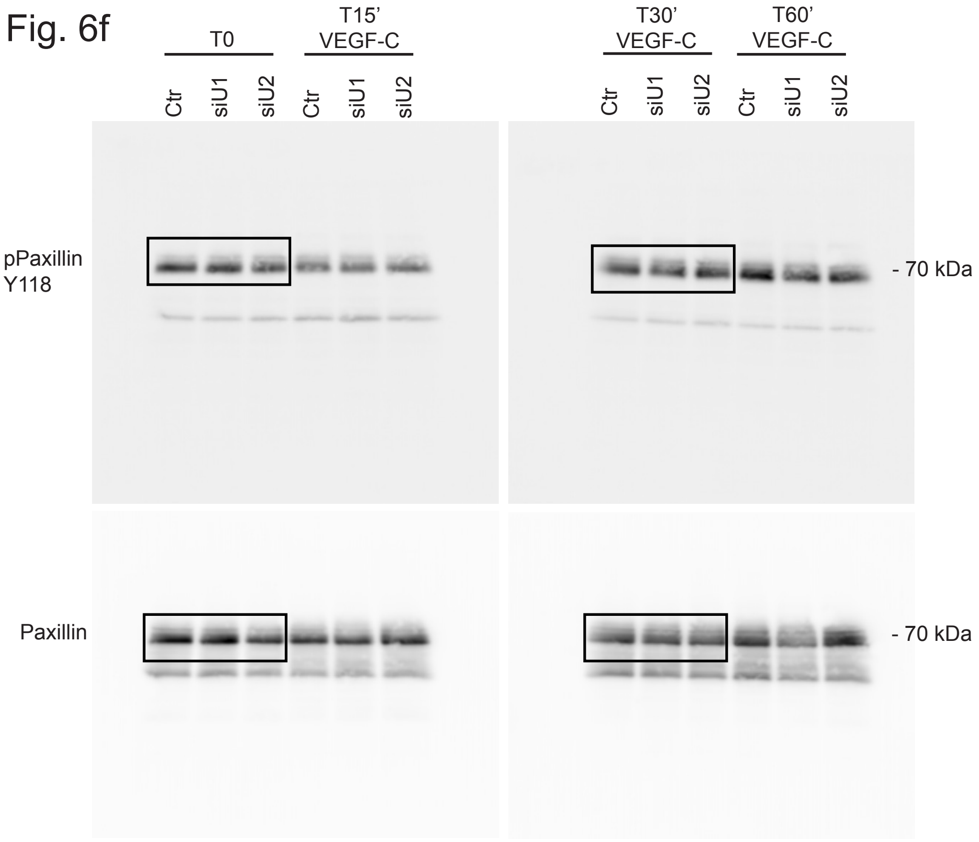

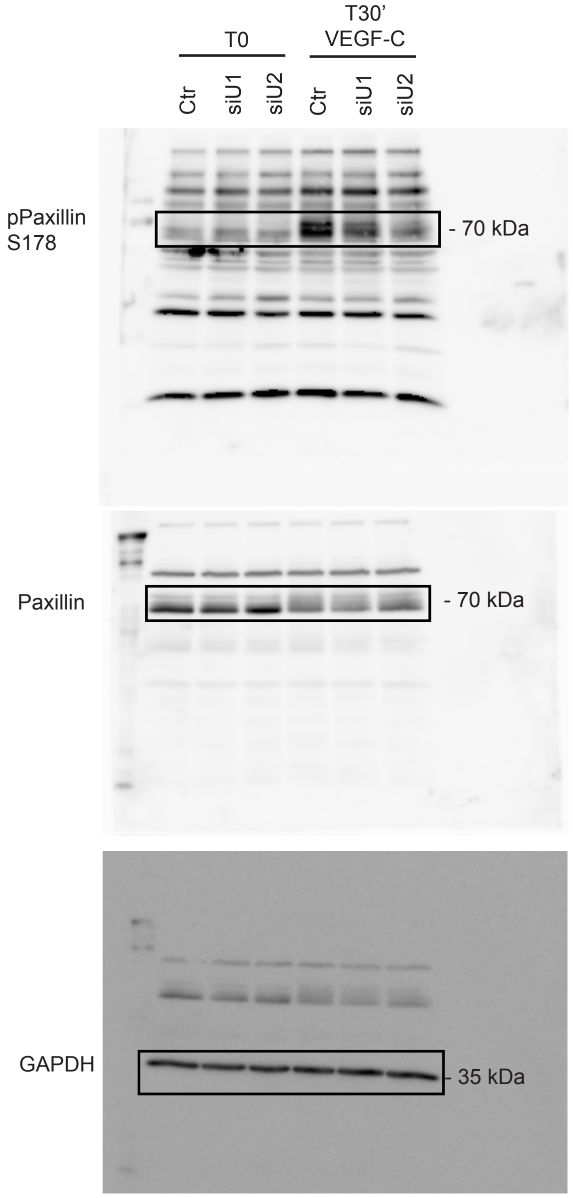

Supplementary Figure 18. Full Western blots. Portions of blots presented in Fig. 6f are indicated.

Fig. 7a

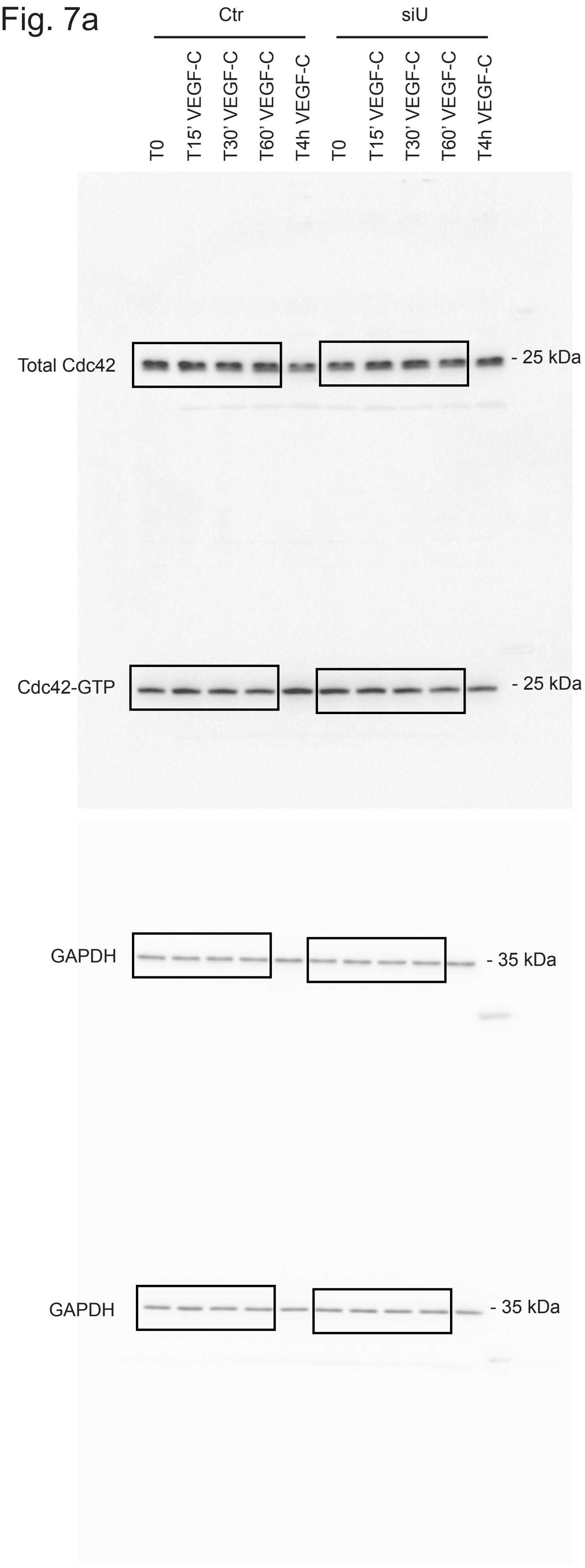

Supplementary Figure 19. Full Western blots. Portions of blots presented in Fig. 7a are indicated.

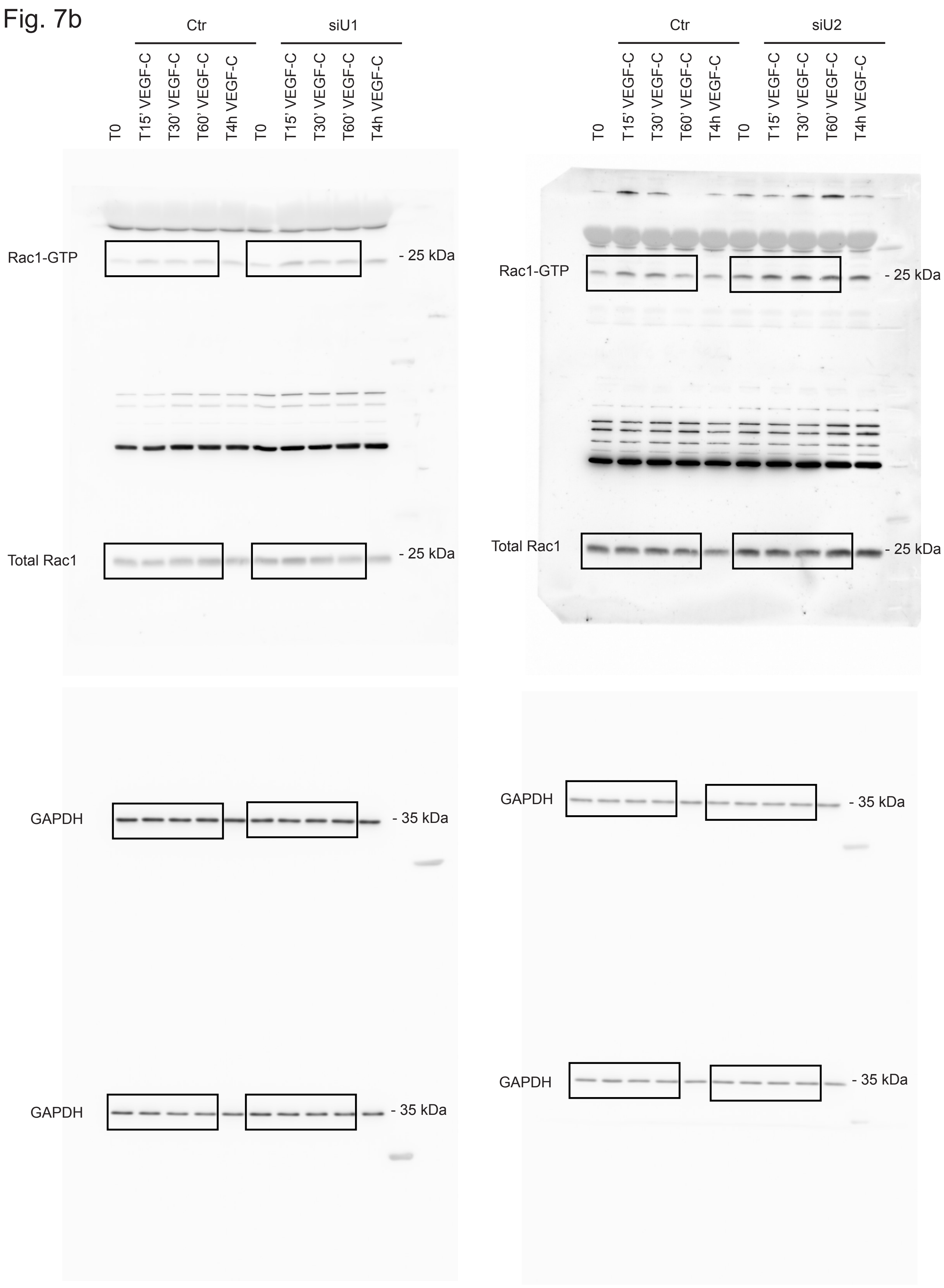

Supplementary Figure 20. Full Western blots. Portions of blots presented in Fig. 7b are indicated.

Supplementary Fig. 6a

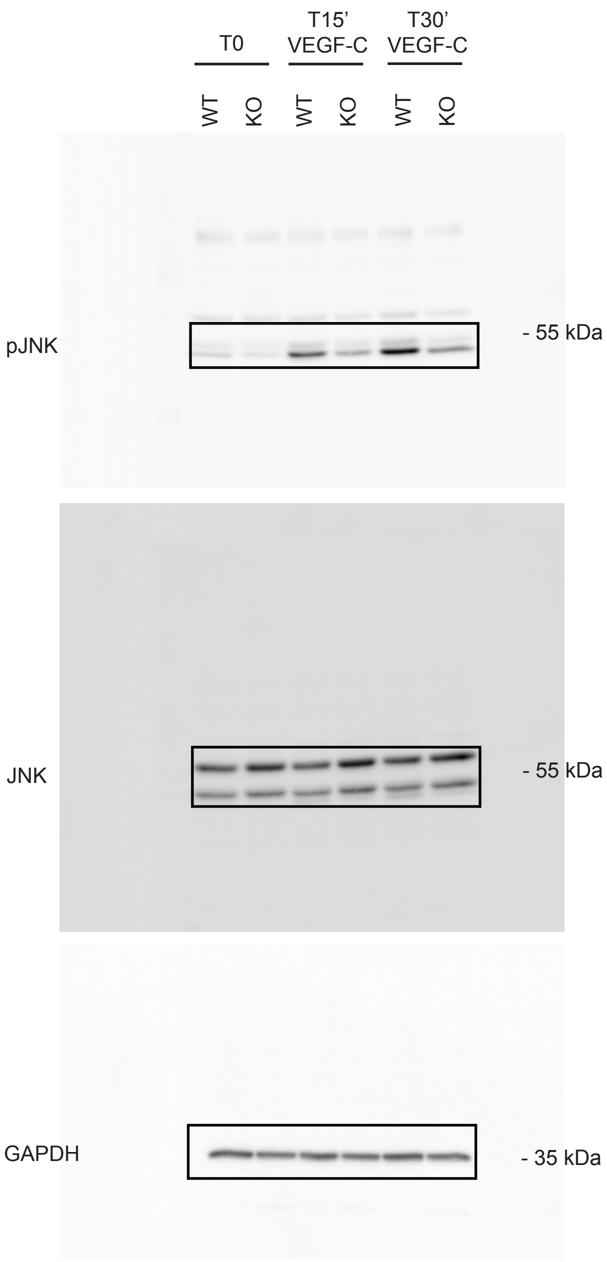

Supplementary Figure 21. Full Western blots. Portions of blots presented in Supplementary fig. 6a are indicated.

Supplementary Reference

1. Detry, B., *et al.* Sunitinib inhibits inflammatory corneal lymphangiogenesis. *Investigative ophthalmology & visual science* **54**, 3082-3093 (2013).
2. Detry, B., *et al.* Matrix metalloproteinase-2 governs lymphatic vessel formation as an interstitial collagenase. *Blood* **119**, 5048-5056 (2012).
